# FAst Segmentation Through SURface Fairing (FASTSURF): A novel semi-automatic hippocampus segmentation method

**DOI:** 10.1371/journal.pone.0210641

**Published:** 2019-01-18

**Authors:** Fabian Bartel, Hugo Vrenken, Marcel van Herk, Michiel de Ruiter, Jose Belderbos, Joost Hulshof, Jan C. de Munck

**Affiliations:** 1 Department of Radiology and Nuclear Medicine, VU University Medical Center, Amsterdam, The Netherlands; 2 Manchester Cancer Research Centre, Division of Cancer Science, School of Medical Sciences, Faculty of Biology, Medicine and Health, University of Manchester, Manchester Academic Health Sciences Centre, Manchester, United Kingdom; 3 Division of Psychosocial Research and Epidemiology, Netherlands Cancer Institute, Amsterdam, The Netherlands; 4 Department of Radiotherapy, Netherlands Cancer Institute, Amsterdam, The Netherlands; 5 Department of Mathematics, VU University Amsterdam, Amsterdam, The Netherlands; McGill University, CANADA

## Abstract

**Objective:**

The objective is to present a proof-of-concept of a semi-automatic method to reduce hippocampus segmentation time on magnetic resonance images (MRI).

**Materials and methods:**

FAst Segmentation Through SURface Fairing (FASTSURF) is based on a surface fairing technique which reconstructs the hippocampus from sparse delineations. To validate FASTSURF, simulations were performed in which sparse delineations extracted from full manual segmentations served as input. On three different datasets with different diagnostic groups, FASTSURF hippocampi were compared to the original segmentations using Jaccard overlap indices and percentage volume differences (PVD). In one data set for which back-to-back scans were available, unbiased estimates of overlap and PVD were obtained. Using longitudinal scans, we compared hippocampal atrophy rates measured by manual, FASTSURF and two automatic segmentations (FreeSurfer and FSL-FIRST).

**Results:**

With only seven input contours, FASTSURF yielded mean Jaccard indices ranging from 72(±4.3)% to 83(±2.6)% and PVDs ranging from 0.02(±2.40)% to 3.2(±3.40)% across the three datasets. Slightly poorer results were obtained for the unbiased analysis, but the performance was still considerably better than both tested automatic methods with only five contours.

**Conclusions:**

FASTSURF segmentations have high accuracy and require only a fraction of the delineation effort of fully manual segmentation. Atrophy rate quantification based on completely manual segmentation is well reproduced by FASTSURF. Therefore, FASTSURF is a promising tool to be implemented in clinical workflow, provided a future prospective validation confirms our findings.

## 1. Introduction

Hippocampus segmentation on structural magnetic resonance images (MRI) is used to monitor morphological hippocampal changes which occur in diseases like Alzheimer’s disease (AD), depression, epilepsy, and schizophrenia [1–4]. Hippocampal volume change is therefore an important biomarker in the quantification of progressive neurodegenerative diseases such as AD or mild cognitive impairment (MCI) [5,6]. In the last few years, hippocampal delineation has also gained importance in radiotherapy during prophylactic cranial irradiation (PCI) aimed at avoiding lung tumour spread to the brain while sparing the hippocampus and reduce neurotoxicity [7–11].

The hippocampus is a small archicortical brain structure which shows limited contrast on structural MRI scans because adjacent structures, such as the amygdala, caudate nucleus and the thalamus typically have similar intensity [12]. This makes hippocampus segmentation a difficult task, regardless of the degree of automation used. Manual segmentation requires extensive training and is labour intensive. Multiple methods have been developed to semi-automatically or fully automatically segment the hippocampus, most of which are discussed in a recent review study by Dill et al [13]. Automatic methods are usually based on deformable models, single-, multiple- or probabilistic-atlases, while semi-automatic methods also involve manual pre- or post-processing. According to Dill et al., the reasons why these methods are still not ready for routine clinical use include the sensitivity of automatic methods to the choice of (patient group dependent) atlases, the computational cost of multiple atlas registration, the lack of validation for different data sets, and the complexity of the required manual pre- and post-processing procedures [13].

Two of the most commonly used automatic segmentation methods in the academic community, FSL-FIRST [14] and FreeSurfer [12,15], have been compared to manual hippocampus segmentation in multiple studies [12,14,16–23,24–31]. Generally, the conclusion was that automatic segmentation methods are promising for population studies, but they need to be further improved for clinical use. A recent study from Mulder and colleagues showed for example that FreeSurfer obtained better atrophy rate reproducibility than manual hippocampus segmentation, but only when FreeSurfer’s outlier segmentations were removed, illustrating that individual subject hippocampus outlining accuracy is not good enough to rely on without expert visual inspection [31].

For hippocampal volume measurements in clinical trials, manual delineation is usually the method of choice [32]. However, even manual segmentations are biased because the precise definition of the hippocampal region varies across laboratories resulting in hippocampal volumes ranging from 2 to 5.3 cm^3^ in studies with different diagnostic groups and outlining protocols [33,34]. It is therefore of crucial importance that manual outlining protocols are standardized as much as possible. Different application areas have developed their own standards. Within neurology, an initiative has been taken to develop a harmonized hippocampal outlining protocol (HarP), by merging hippocampal boundary definitions from different outlining protocols [34–36]. Within radiotherapy, due to the integration of hippocampal avoidance treatment plans in radiotherapy, another hippocampus outlining protocol has been developed by the radiotherapy oncology group (RTOG, [37]). These protocols differ in terms of the definitions of boundaries and the anatomical orientation of the images used for outlining.

Manual segmentation protocols are mainly focussed on reproducibility and standardization, whereas the delineation efficiency is greatly ignored. Typically, it requires one to two hours to segment a complete hippocampus pair. With this study, we present a novel semi-automatic hippocampus segmentation method: FAst Segmentation Through SURface Fairing (FASTSURF). The method is based on mesh processing techniques, is computationally inexpensive and does not require a priori knowledge such as atlases or models. The underlying idea of FASTSURF is that the slice to slice changes of hippocampal cross-sections are generally small. Therefore, using certain smoothness constraints, the hippocampal shape can be reconstructed from a few manually delineated cross-sections. In this study, these few delineated cross-sections are simulated from full manual delineations. FASTSURF is then validated by comparing it to these fully manual segmentations, using different datasets from different diagnostic groups. Because the underlying principle is applicable to different outlining protocols, it is tested for the HarP and RTOG protocols and for a protocol from Jack et al. [38]. Finally, a comparison is made with automatically segmented hippocampi using FreeSurfer [12,15] and FSL-FIRST [14].

## 2. Materials and methods

### 2.1. Datasets and MRI acquisition

We used three different datasets to validate our method, one dataset with subjects from the Netherlands Cancer Institute–Antoni van Leeuwenhoek (NKI-AvL) hospital in Amsterdam, the Netherlands (Dataset 1, described below) and two different datasets from the Alzheimer’s Disease Neuroimaging Initiative (ADNI) database (Datasets 2 and 3, described below). Datasets 2 and 3 used in the preparation of this article were obtained from the ADNI database (adni.loni.usc.edu). The ADNI was launched in 2003 as a public-private partnership, led by Principal Investigator Michael W. Weiner, MD.

#### 2.1.1. Dataset 1

Dataset 1 is a subset of data from a multicentre phase III trial in which patients with small cell lung cancer (SCLC) receive either standard PCI treatment or PCI treatment with hippocampal avoidance (Clinical trials.gov identifier: NCT01780675). MRI data were anonymously accessed and collected at the NKI-AvL. The imaging protocol was the same as in the ADNI GO study. Sagittal 3D T1-weighted MRI were acquired with a magnetization prepared rapid acquisition gradient echo (MPRAGE) sequence using a 3T Philips Achieva with an eight channel head coil. For all MRIs, pixels in-plane were 1mm^2^ with a slice thickness of 1.2mm. Data and hippocampus delineations of 12 patients who received PCI with hippocampal avoidance were collected.

#### 2.1.2. Dataset 2

Dataset 2 was taken from the ADNI database with images and training labels of 135 subjects of different diagnostic groups, acquired with two different MRI scanner field strengths of 1.5T and 3T using various MRI scanner vendors (Philips, Siemens and GE). Sagittal 3D T1 weighted MPRAGE images were acquired for 44 healthy control (CTRL), 46 MCI and 45 AD subjects. In-plane pixel sizes ranged from 0.86mm to 1.25mm and slice thickness was 1.2mm. In [39] a detailed description of the imaging protocol is given.

#### 2.1.3. Dataset 3

The third dataset is the same ADNI dataset as was used in [31] and [40]. The dataset consists of 80 subjects, 20 CTRL, 40 MCI and 20 AD subjects. For each subject, four volumetric MRI scans were collected. Two MRI back-to-back (BTB) scans were acquired at time-point baseline (BL-A and BL-B) and two MRI BTB scans one year later (M12-A and M12-B). The BTB scans were acquired in a single session with just a few seconds between acquisitions but processed independently. The BL scans were acquired between September 2005 and August 2007. Sagittal 3D T1 weighted MPRAGE images were acquired at 1.5T field scanners from different vendors (Philips, Siemens and GE). The four scans for each subject were acquired with the same MRI scanner and protocol. In-plane pixel sizes ranged from 0.93mm to 1.2mm and slice thickness was 1.2mm. Images were not further processed other than the default scanner corrections and visual inspection of each scan ensured good quality. In [41] a more detailed description of the MRI acquisition can be found.

### 2.2. Manual and automatic hippocampus segmentation

#### 2.2.1. Manual hippocampus segmentation for dataset 1

The clinical Dataset 1 was delineated using the RTOG protocol for hippocampal sparing [37]. Using a rigid body registration, MRIs were registered to treatment-planning CTs with 1mm slices thickness and in-plane pixel sizes varying between 0.6mm and 0.7mm. Hippocampi were delineated on these resliced axial MRI slices. The most inferior slice to delineate the hippocampus is defined to be the slice on which the temporal horn appears next to the lateral ventricle. Hippocampal grey matter is segmented from the anterior to the superior direction while avoiding the fimbria. The anterior boundary is defined by the temporal horn and the amygdala, the medial boundary by the uncus. In postero-cranial direction the medial boundary is formed by the lateral edge of the quadrageminal cistern. On the last slices in postero-cranial direction the hippocampus is located antero-medially to the atrium of the lateral ventricle and hippocampus segmentation ends when the crux of the fornix emerges. The average number of slices on which the hippocampus was outlined is 21.1 (see Table 1).

**Table 1:** For all subjects of each dataset, the average number of slices on which the hippocampus was segmented is shown.

Dataset	N. of Slc.	
	Mean	STD
1	21.1	±4.14
2	37.3	±3.74
3	19.9	±1.48

*N*. *of Slc*. Number of Slices, *STD* Standard Deviation

#### 2.2.2. Manual hippocampus segmentation for dataset 2

Scans of dataset 2 were outlined using the EADC-ADNI Harmonized Protocol for Hippocampal Segmentation (HarP) described in [35] and segmentation files were obtained from the HarP project’s website (http://www.hippocampal-protocol.net/). Briefly, MRIs were aligned along the anterior and posterior commissures of the brain (AC-PC line) by using a rigid body registration to the MNI ICBM152 template (International Consortium for Brain Mapping) with 1x1x1mm voxel dimensions and images were resampled with trilinear interpolation. The most posterior slice where the hippocampus is segmented is defined to be the slice on which a small ovoid grey matter mass is visible close to the lateral ventricle. The most anterior slice to outline the hippocampus is defined to be the slice on which the alveus can be seen below the amygdala. For detailed boundary descriptions and figures we refer the reader to the HarP literature [34–36].

#### 2.2.3. Manual hippocampus segmentation for dataset 3

Scans of (ADNI) Dataset 3 were segmented at the Image Analysis Center (IAC, VU University Medical Center (VUmc) Amsterdam) using a segmentation protocol from [38], previously described in [31,38,42]. For all subjects the BL MRI scans were reformatted in a plane perpendicular to the long axis of the left hippocampus resulting in a pseudo coronal orientation. Sinc interpolation was used, slice thickness was 2mm, and the original in-plane resolution was maintained. M12 scans were rigidly registered to BL scans, again using sinc interpolation. All hippocampi were segmented by a single well-trained expert of the IAC using in-house developed software (Show_Images 3.7.1.0). Following the IAC protocol, BL segmentations were shown alongside M12 scans when M12 scans were segmented. However, the technician was blinded to the diagnosis and BTB scans were given in random order.

The hippocampal formation consists of the Ammon’s horn, dentate gyrus, alveus and fimbria and the subiculum. When detecting the total length of the crux of the fornix the most posterior slice to outline the hippocampus can be seen. The inferior boundary is formed by the subiculum and the parahippocampal gyrus and the superior boundary by the CSF of the temporal horn and the alveus. The lateral border is defined by the CSF and the temporal horn and the alveus, while the medial border is defined by the CSF in the cisterna ambiens and the transverse fissure. The most anterior slice on which the hippocampus is outlined, is defined to be the slice on which the hippocampus appears alongside the amygdala and CSF appears on the medial side of the hippocampus.

#### 2.2.4. FSL-FIRST hippocampus segmentation (only dataset 3)

FSL-FIRST is an automatic segmentation tool based on deformable models. Details are described in [43] and [14]. Briefly, with a set of manual segmented hippocampi from the Center for Morphometric Analysis (CMA), Massachusetts General Hospital (MGH) Boston, shape and appearance models were constructed. For this, a point distribution model was created using parameterized surface meshes created from the manual segmentations taking into account the intensity around the tissue border. To segment a new MRI, FSL-FIRST uses intensity values from the MRI and searches through linear combinations of shape variation modes to find the most probable shape. Before segmentation, FSL-FIRST performs a two-stage affine registration to MNI152 standard space at 1mm resolution. Then, by using FAST voxel-wise segmentation software [44] the hippocampus mesh is converted to a labelled image. We used FSL-FIRST v.5.0.4 with the script command run_first_all. The voxel-wise hippocampal labels produced by FSL-FIRST are in native MRI scan space.

#### 2.2.5. FreeSurfer hippocampus segmentation (only dataset 3)

FreeSurfer automatic segmentation for subcortical structures involves multiple steps and is described in detail in [12]. First, MRI scans are transformed to a conformed 1mm^3^ 256^3^ space. FreeSurfer performs bias-field correction and intensity normalization, and strips the skull to transform an atlas to the brain. Voxels are assigned to subcortical structures using prior probabilistic intensity and tissue class information.

To obtain FreeSurfers hippocampus segmentation FreeSurfer version 5.3 was used with the longitudinal stream for longitudinal data (Dataset 3) and cross-sectional stream for cross-sectional data. FreeSurfer’s voxelwise hippocampal labels from the cross-sectional and longitudinal stream were converted back to the native MR image space using the procedure provided by FreeSurfer (mri_label2vol).

Like FSL-FIRST, FreeSurfer uses the CMA segmentation scheme for subcortical segmentation. The segmentation protocol can be found on their website (http://freesurfer.net/fswiki/CMA). The substructures of this outlining protocol are similar to the substructures mentioned in the outlining protocol from [38] of dataset 3: dentate gyrus, cornu ammonis, subiculum, fimbria and alveus.

#### 2.2.6. Surface reconstruction and volumetric analysis

We converted all voxel-wise hippocampal labels to meshes using the marching cube algorithm. To reduce interpolation errors as much as possible, all volumes and overlap indices were computed from these meshes after applying the appropriate registration transformation as described previously in [40].

Using IBM SPSS Statistics for Windows v. 22 Armonk, NY: IBM Corp we performed a one-way repeated measures ANOVA to determine volumetric differences in dataset 3 between manual and automatic segmentation methods. A post hoc analysis was performed after Bonferroni’s correction.

### 2.3. FASTSURF

#### 2.3.1. Theory

**Fig 1:**
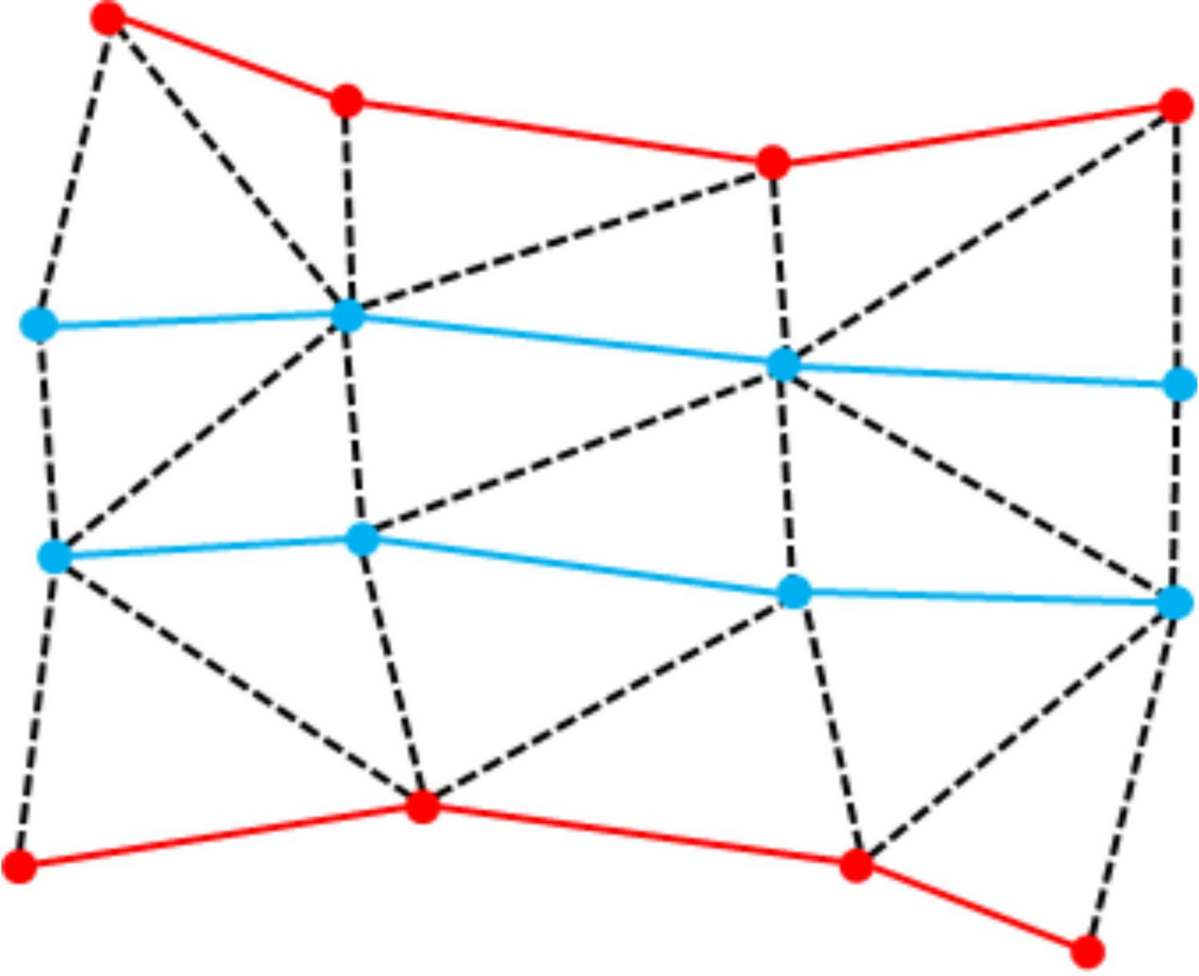
Schematic representation of delineated and intermediate contours. Delineated contours are represented in red with known point positions, intermediate contours are represented in blue with unknown point positions. The black dashed lines complete the triangulated mesh.

FASTSURF is based on sparse hippocampus contouring, with the missing contours computed automatically, under the constraint that contours of the most extreme slices of the hippocampus are available. We define a *contour* as a closed tracing of the hippocampus perimeter on a single slice. Delineated contours are connected by constructing a triangular mesh of which some nodes correspond to the delineated points and the remaining points move to intermediate positions determined by applying certain smoothness constraints. This technique is known as surface fairing [45]. A schematic representation of delineated and intermediate contours is represented in Fig 1.

The mesh so obtained can be considered as a graph, in which every vertex is connected to a set of neighbours. Then, given the connectivity graph, the discrete Laplacian is defined as follows:

$$L_{n,m}=\left\{ \begin{array}{cc} 1 & if\;n=m\\ \frac{-1}{N_{Neighbours}(v_{n})} & v_{n}is\;adjacent\;to\;v_{m}\\ 0 & otherwise \end{array}\right.$$ (1)

where the indices *n* and *m* refer to the mesh vertices and *N*_*Neighbours*_(*v*_*n*_) is the number of neighbours of vertex *v*_*n*_. When all the edges are interpreted as springs with a fixed spring constant and when a net force balance of zero is imposed on each vertex, both at known and unknown vertices, optimal vertex positions are obtained by setting

$$\sum_{m}L_{n,m}x_{m}=\sum_{m}L_{n,m}y_{m}=\sum_{m}L_{n,m}z_{m}=0$$ (2)

where ***x***, ***y***, and ***z*** are vectors of the *x*-, *y*- and *z*-coordinates of all mesh vertices. Coordinates of the unknown intermediate vertices can be found by moving all known points to the right hand side of these equations and by solving the three sparse systems of equations, for which we used the iterative bi-conjugate gradient method [46]. Finding the intermediate vertices with these equations would lead to a surface of minimum area, or *minimal surface*, and no penalty is put on the increased curvature at the delineated points. When minimizing the curvature instead of surface area, a *thin-plate surface* is obtained, requiring only a minor modification of the equations. Translating continuous curvature minimization functions to a discrete triangle mesh [45] leads to linear bi-Laplacian systems:

$$\sum_{m}{L_{n,m}}^{2}x_{m}=\sum_{m}{L_{n,m}}^{2}y_{m}=\sum_{m}{L_{n,m}}^{2}z_{m}=0$$ (3)

This approach has similarities to spline interpolation, in which continuity of a function and its derivatives is enforced at all edges and nodes and the interpolating triangles are curved. However, in our approach the triangles are flat and a numerical approximation of the minimum surface curvature, resulting to simpler and probably faster computations.

**Fig 2:**
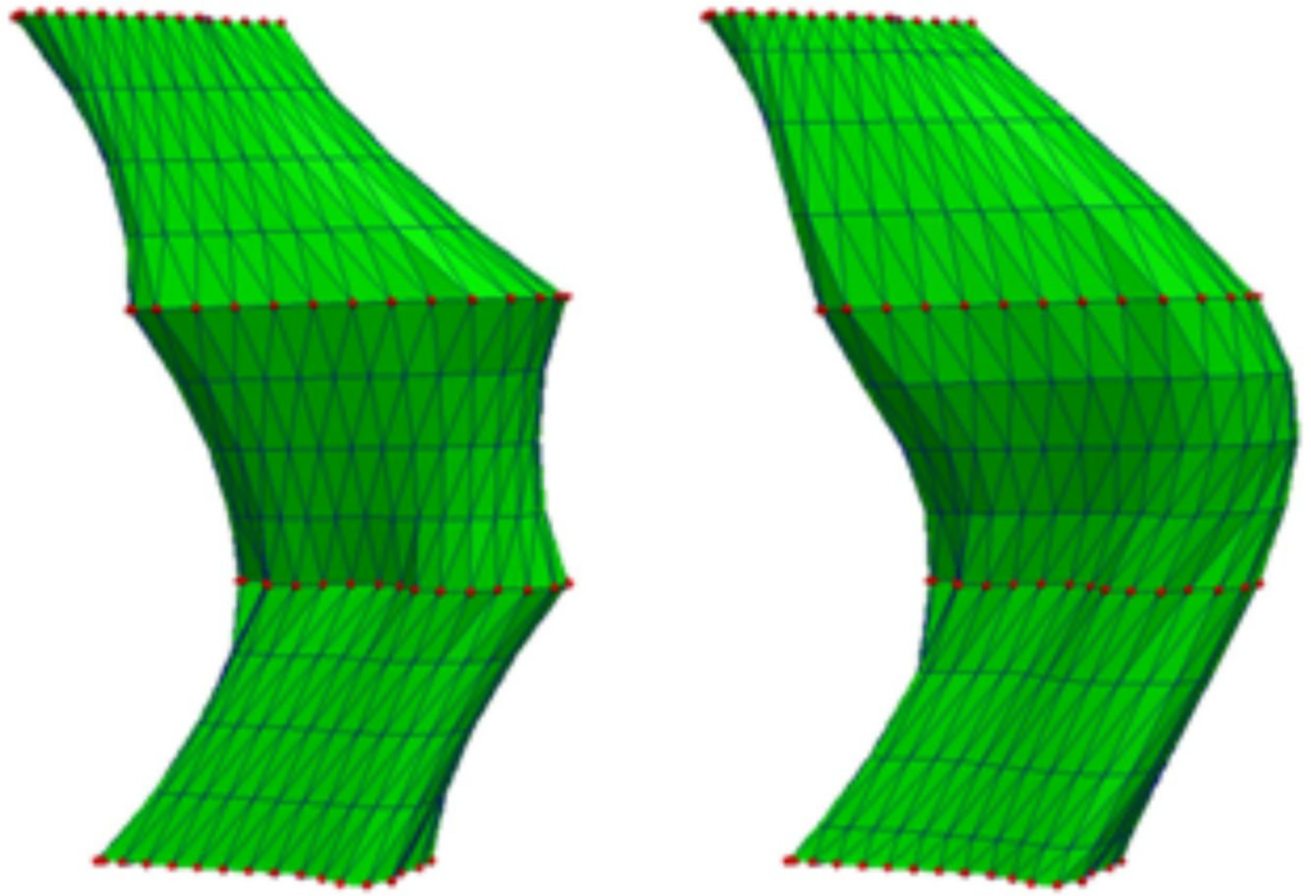
Surface reconstructions by solving a sparse system of equations with known points denoted in red. Left: Surface reconstruction using Laplacian operator. Right: Surface reconstruction using bi-Laplacian operator (FASTSURF).

An example showing the difference between a Laplacian and bi-Laplacian solution is presented in Fig 2. In the remainder of this paper we use the term “FASTSURF segmentation” to denote sparse hippocampal outlines which were completed by solving the bi-Laplacian systems.

#### 2.3.2. Simulation of sparse delineation

To demonstrate the proof of concept, we simulated sparse delineations to evaluate FASTSURF segmentation. Manually delineated hippocampus segmentations were converted to 3D meshes from which we extracted a number of contours at regular intervals. The contours were extracted in the same direction in which the hippocampus was segmented, i.e. for dataset 1 the contours were extracted in axial direction and for dataset 2 and 3 in (pseudo) coronal direction. Then, we linearly interpolated a predefined number of points on each contour and replaced the original contour points with the interpolated ones to obtain the same number of points equally distributed on each contour. Then, as a first approximation, contours were connected by straight lines and intermediate contours were created parallel to the simulated contours with the same predefined number of points. A regular triangular mesh was defined, connecting the original and intermediate points. Finally, by solving the bi-Laplacian systems, we obtained new vertex positions for the points of the intermediate contours and updated the contours resulting in a smooth surface mesh.

#### 2.3.3. Comparison of FASTSURF segmentation to manual and automatic segmentation

We used overlap indices and percentage volume difference measures to compare FASTSURF segmentation with completely manual hippocampus segmentation. The Jaccard index was computed directly from the surface meshes by adopting a fine regular grid enclosing the two surfaces. The Jaccard index was approximated by:

$$Jacc(A,B)\approx \frac{N_{A\cap B}}{N_{A\cup B}},$$ (4)

where *N*_*A*∩*B*_ and *N*_*A*∪*B*_ are the number of grid points inside the cross section and the union of both surfaces, respectively. The Jaccard index is directly related to the Dice overlap index (D = 2J/(J+1)). Hippocampus meshes from different MRI scans generally are in different spaces. Before applying (4), we first performed a rigid body co-registration of the BTB MRI scans with FSL-FLIRT [47,48] and applied the obtained registration parameters on the mesh points of the hippocampi meshes to bring the meshes into the same space. Cross-sectional percentage volume difference was computed using:

$$PVD(A,B)=2\frac{V_{A}-V_{B}}{V_{A}+V_{B}}*100,$$ (5)

and longitudinal percentage volume change was defined by:

$$PVC(A,B)=\frac{V_{A}-V_{B}}{V_{A}}*100,$$ (6)

with *V*_*A*_ being the volume of object *A* and similarly *V*_*B*_.

For dataset 3 we obtained FSL-FIRST and FreeSurfer hippocampus segmentations and compared these segmentations to manual and FASTSURF segmentations. Using the longitudinal BTB scans’ hippocampus segmentations of dataset 3, we computed atrophy rates as defined in (6) using BL and M12 scans.

**Fig 3:**
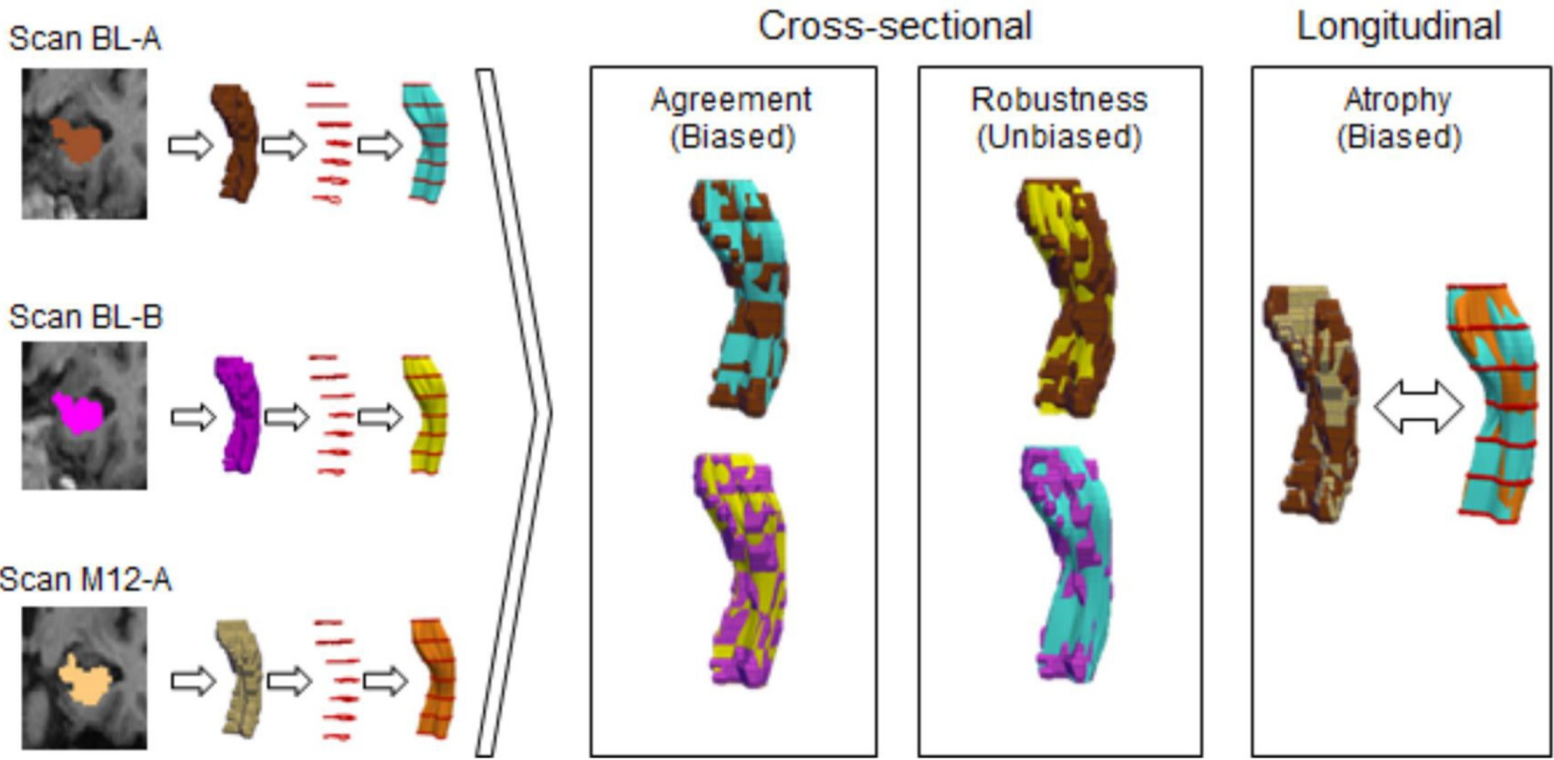
Schematic illustration of comparisons performed using the ADNI BTB dataset (Dataset 3). On the left-hand side labelled hippocampus segmentations from different time-points are converted to meshes, contours are extracted and hippocampi are reconstructed using FASTSURF. The colours help to visually differentiate between manual and FASTSURF segmentations. The boxes on the right-hand side illustrate the comparisons performed for this particular dataset.

When comparing FASTSURF segmentations to manually outlined hippocampus segmentations, results will be biased because the input contours of the simulated sparse delineation are taken from points very close to the fully outlined manual segmentations. Using the BTB scans of dataset 3, we overcome this bias by comparing independent manually outlined hippocampus segmentations from the A scans with FASTSURF segmentations from the B scans, and vice versa. Having A and B scans from both BL and M12, this comparison can be performed twice for each subject, which strengthens the statistical analysis. Using this comparison, we were also able to quantify the bias. Without the availability of real segmented sparse contours, we consider this comparison as an adequate unbiased test of our method’s performance. In the remainder of this manuscript we call this “robustness analysis”. The robustness analysis was performed for both manually and automatically segmented hippocampi. An unbiased atrophy analysis could not be performed with the manual segmentations of this dataset, because the hippocampi on the M12-A and M12-B scans were segmented alongside the corresponding scans and segmentations of the BL time point, i.e. BL-A and BL-B respectively, to determine longitudinal volume change. Therefore, the A and B scans cannot be fairly interchanged for this type of analysis. Agreement, robustness and atrophy comparisons are illustrated in Fig 3 with coloured 3D meshes representing manual and FASTSURF segmentations from different time-points.

**Fig 4:**
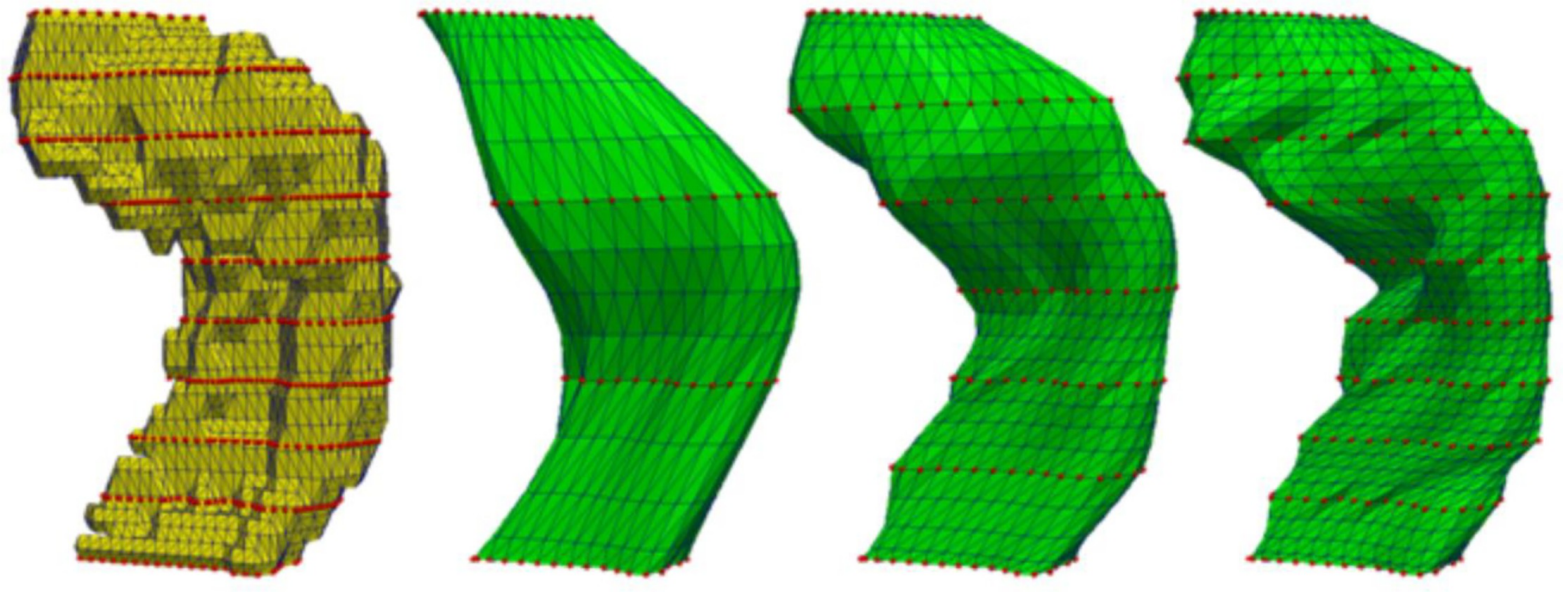
FASTSURF segmentation (green) using different numbers of contours (4, 7 and 10 contours) from a full manual segmentation (yellow). The red dots represent input contours.

All measurements were performed in groups (CTRL, MCI and AD). Furthermore, we tested FASTSURF using different numbers of contours, with a minimum number of four contours. We aimed to reduce the number of contours at least by half, thus for dataset 1 the number of contours used for hippocampus reconstruction ranged from 4–10, for dataset 2 it ranged from 4–18 and for dataset 3 we used a range of 4–10. An example using FASTSURF with different numbers of contours is presented in Fig 4.

#### 2.3.4. Parameter tuning

Parameter refinement and bug-testing for FASTSURF was performed on 10 randomly chosen MCI subjects’ hippocampal segmentations from Dataset 3, using both BTB scans. These 10 subjects’ hippocampal segmentations were excluded in our final analysis. We extracted 10 contours from these subjects’ segmentations and tested the effects of the number of intermediate contours and the number of points used in the triangulation step for each contour. Using the BTB scans’ segmented hippocampi, we performed agreement and robustness analysis for FASTSURF segmentations with manual hippocampal segmentations. Table 2 shows results for optimizing the number of intermediate contours (using 50 points per contour) and Table 3 shows the test results for optimizing the number of points on each contour. In both tables means and standard deviations (STD) of resulting Jaccard indices and PVDs are presented.

**Table 2:** From 10 randomly chosen MCI subjects’ BTB hippocampus segmentations, 10 contours were extracted to simulate delineations and the number of intermediate contours between subsequent delineation simulations was varied. Agreement and robustness were determined as described in the main text.

No. of Int. Cont.	Agreement				Robustness			
	Jaccard		PVD		Jaccard		PVD	
	Mean	STD	Mean	STD	Mean	STD	Mean	STD
1	.853	.0240	3.396	2.2989	.778	.0239	-3.395	4.2504
2	.851	.0241	2.983	2.0606	.777	.0239	-2.982	4.1015
3	.849	.0242	2.974	1.9974	.776	.0240	-2.973	4.0589
4	.845	.0242	3.169	1.9991	.775	.0241	-3.168	4.0546
5	.841	.0245	3.511	2.0312	.772	.0240	-3.510	4.0688
6	.836	.0246	3.977	2.0851	.770	.0240	-3.976	4.0973

*No*. *of Int*. *Cont*. Number of Intermediate Contours, *PVD* Percentage Volume Difference, *STD* Standard Deviation

**Table 3:** From 10 randomly chosen MCI subjects’ BTB hippocampus segmentations, 10 contours simulated and, using three intermediate contours, the number of points for each contour was varied. Agreement and robustness were determined as described in the main text.

No. of Pnts.	Agreement				Robustness			
	Jaccard		PVD		Jaccard		PVD	
	Mean	STD	Mean	STD	Mean	STD	Mean	STD
10	.561	.0253	44.900	3.9171	.539	.0234	-44.882	5.5727
50	.849	.0242	2.974	1.9974	.776	.0240	-2.973	4.0589
100	.856	.0241	2.139	1.8827	.777	.0237	-2.138	4.0104
150	.857	.0247	2.002	1.8696	.776	.0237	-2.001	4.0094
200	.856	.0247	1.960	1.8667	.776	.0238	-1.960	4.0092
250	.856	.0247	1.941	1.8622	.776	.0234	-1.941	4.0087
300	.856	.0246	1.932	1.8645	.776	.0235	-1.932	4.0110
350	.857	.0245	1.927	1.8649	.776	.0235	-1.926	4.0122
400	.857	.0246	1.923	1.8624	.776	.0235	-1.922	4.0112

*No*. *of Pnts*. Number of Points, *PVD* Percentage Volume Difference, *STD* Standard Deviation

Table 2 shows that agreement and robustness hardly depend on number of intermediate contours, but three intermediate contours give best results. With three intermediate contours we optimized the number of points for each contour. Table 3 shows that Jaccard indices increase as a function of this number, until about 100 points per contour. PVDs slightly get closer to zero with increasing number of points per contour, but computational times also increase. Therefore, we chose to perform our final analysis with 100 points per contour and three intermediate contours.

## 3. Results

Hippocampal volumes for specific groups are presented in Table 4, in which for all datasets left and right hippocampal volumes were grouped together, and for dataset 3 hippocampal volumes from all time-points were grouped together. Because of the violation of sphericity, the univariate repeated measures ANOVA was Greenhouse-Geisser corrected. Mean hippocampal volumes showed a significant dependence on method (BL left p = 0.000459, BL right p = 1.4E^-10^, M12 left p = 0.000002, M12 right p = 6.3E^-14^). The post hoc analysis showed that manual BL left and right did not significantly differ from FreeSurfer’s hippocampal volumes (p = 0.341 and p = 0.070), but they were significantly different from FSL-FIRST volumes (p = 0.000139 and p = 1.8E^-10^). FSL-FIRST BL left was not significantly different from FreeSurfers’ BL left but right hippocampal volumes were significantly different (p = 0.070 and p = 0.000009). Manual M12 left and right hippocampal volumes were significantly different from both FSL-FIRST and FreeSurfers’ volumes (Manual vs. FSL-FIRST: p = 8.9E^-8^ (left) and p = 8.7E^-14^ (right); Manual vs. FreeSurfer: p = 0.039 (left) and p = 0.011(right)). FSL-FIRST M12 left and right hippocampal volumes were significantly different than FreeSurfers’ volumes (p = 0.030 (left) and p = 0.000003 (right)).

**Table 4:** Volumes extracted from hippocampal meshes. Volumes are shown in mm^3^. Left and right hippocampal volume was grouped together. For dataset 3 hippocampi from all time-points were grouped together.

Group	Dataset 1	Dataset 2	Dataset 3 Manual	Dataset 3 FSL-FIRST	Dataset 3 FreeSurfer
SCLC	2227.0(±502.38)				
CTRL		3108.1(±512.7)	3351.5(±405.52)	3537.2(±452.51)	3555.4(±485.45)
MCI		2650.8(±467.8)	3082.7(±482.70)	3297.6(±540.39)	3158.3(±565.66)
AD		2364.7(±528.94)	2760.1(±572.06)	2898.0(±530.05)	2703.2(±640.09)

*SCLC* Small Cell Lung Cancer, *CTRL* Control, *MCI* Mild cognitive impairment, *AD* Alzheimer Disease

**Fig 5:**
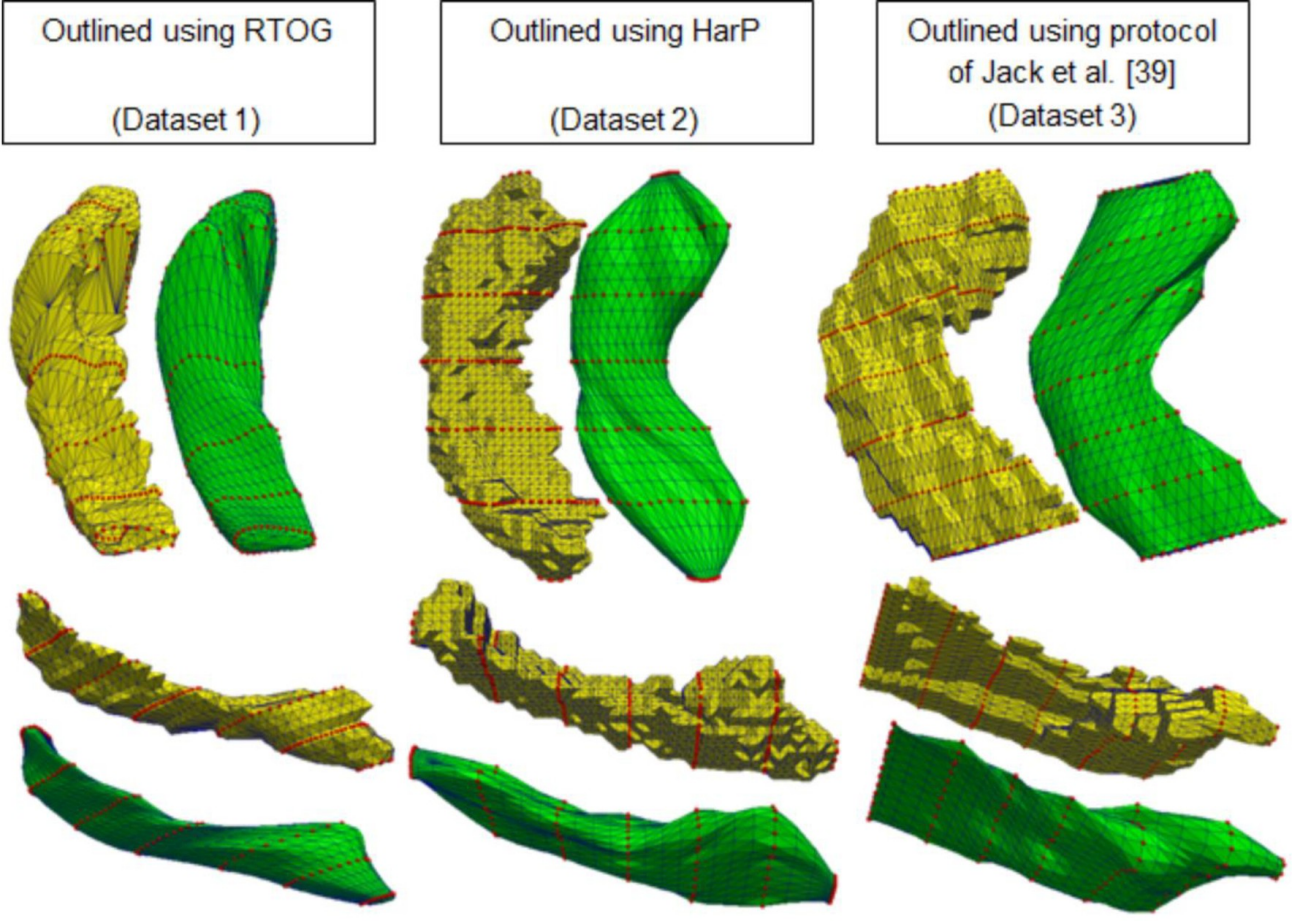
Differences between manual hippocampus segmentations and FASTSURF segmentations for the three different manual outlining protocols. Yellow represents manual hippocampus segmentations and green the FASTSURF segmentation using seven contours. Hippocampi are from different subjects randomly chosen from each dataset. Top and bottom are the same hippocampi shown in different orientation in 3D space.

The volumes differ between datasets due to different operational procedures and protocols. For instance, hippocampi outlined on resampled MRI of dataset 2 generally have more contours than hippocampi from the other datasets and hippocampi from dataset 1 are outlined in axial direction. Fig 5 illustrates these differences by presenting surface renderings of one example from each dataset for manual and FASTSURF hippocampi using seven contours.

### 3.1. Results for dataset 1

**Fig 6:**
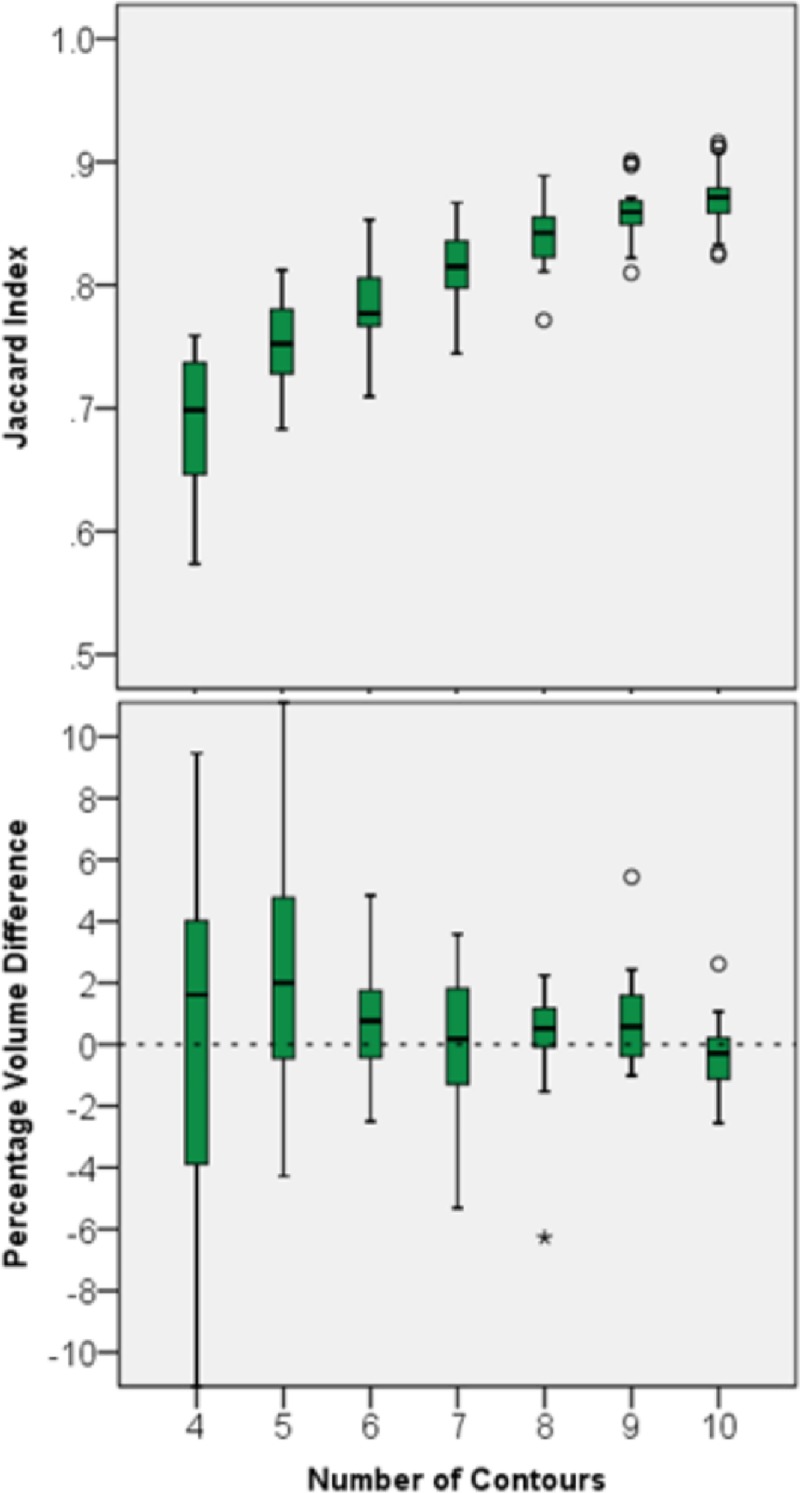
Agreement between manual and FASTSURF segmentations using a varying number of input contours for Dataset 1. Top boxplot shows Jaccard indices and the bottom boxplot PVDs. The small circle and the star sign are outliers defined by the SPSS software, with the star sign being a “far out” outlier.

Hippocampi in dataset 1 were outlined using the RTOG protocol and FASTSURF segmentations were generated using 4 to 10 contours. Jaccard indices and PVDs are plotted in boxplots in Fig 6. S1 Table displays all corresponding mean and standard deviations for Fig 6. As expected, with increasing number of contours Jaccard indices increase, and PVDs get close to zero. It should be noted, ignoring the bias in these results for now, that with only five contours a Jaccard index higher than 0.67 (equivalent to a Dice overlap of 0.8) is reached. This is considered as good accuracy for small structures as the hippocampus [12,13]. PVDs for six or more contours are relatively consistent. Five to six contours would mean a theoretical time reduction to approximately one fourth of the original time needed, considering that the mean number of hippocampal contours for this dataset is ~21.

### 3.2. Results for dataset 2

**Fig 7:**
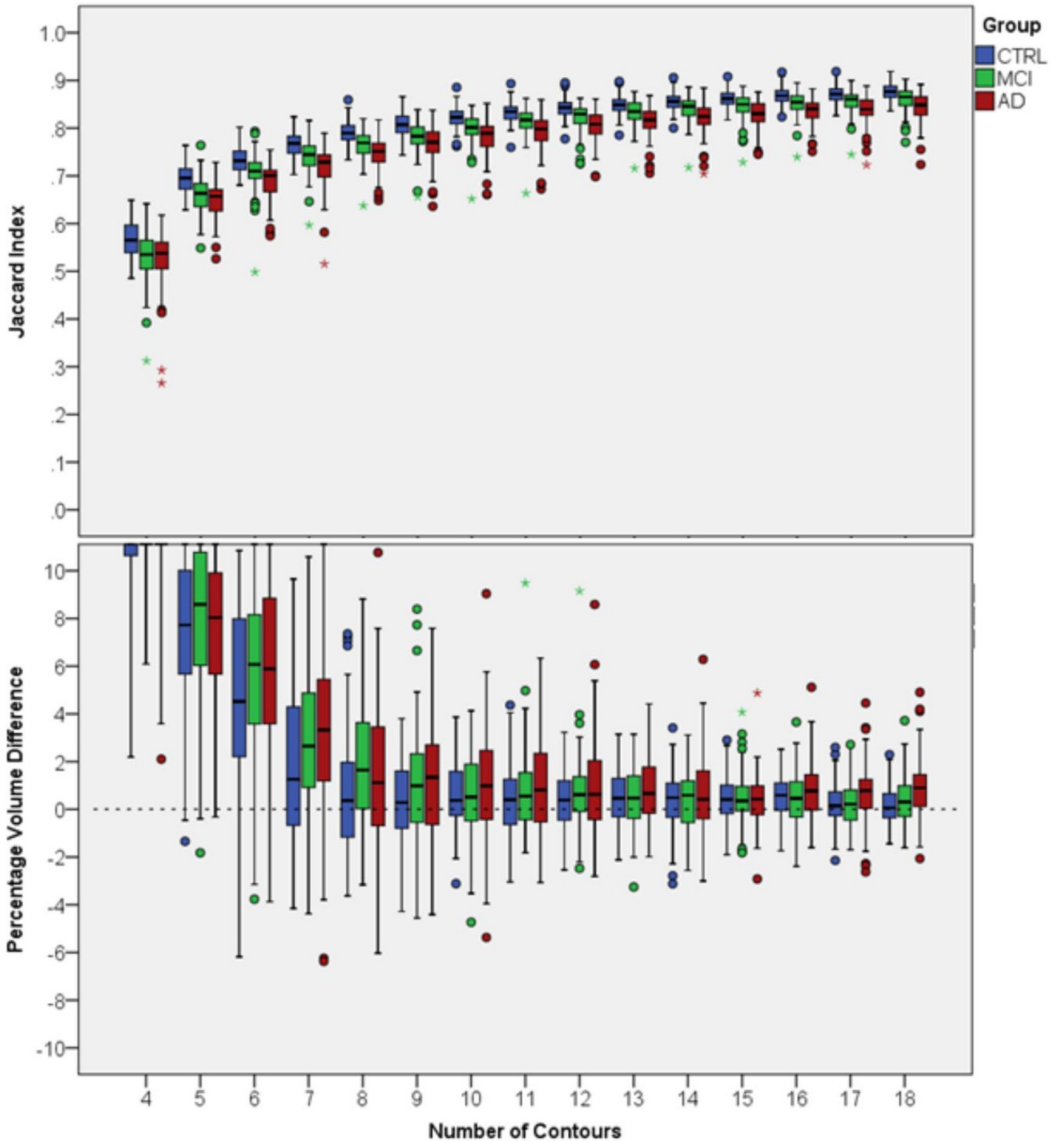
Agreement of FASTSURF with manual hippocampus segmentations for Dataset 2. Top boxplot shows Jaccard indices and the bottom boxplot PVDs. Both plots are split into three panels, each representing one group (CTRL, MCI and AD). The small circle and the star sign are outliers defined by the SPSS software, with the star sign being a “far out” outlier.

**Fig 8:**
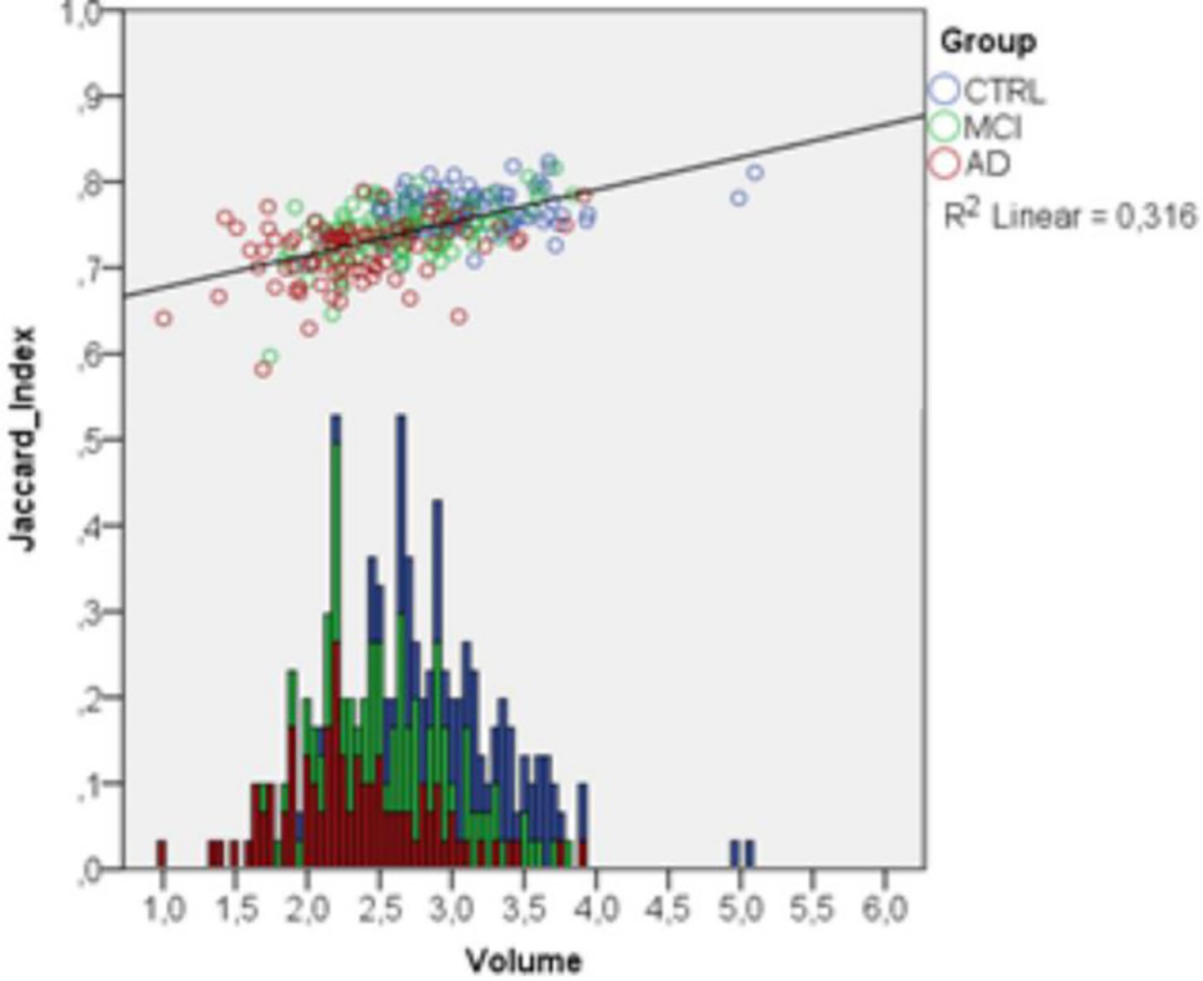
Relation between volumes in cm^3^ of fully manual hippocampus segmentation and Jaccard indices obtained from the comparison of FASTSURF segmentation using seven contours and the manual segmentations. At the bottom the volume histogram is plotted to show volume frequencies for specific groups.

For dataset 2 we performed a similar analysis separately for each patient group. Fig 7 shows overlap indices and PVDs of FASTSURF and manual segmentations per group as a function of the number of input contours. For enhanced visibility, we scaled the PVD boxplot cutting off larger outliers for four to six contours, but all mean and standard deviations can be found in the S2 Table. With eight or more contours, Jaccard indices above 0.67 and relatively low PVDs were obtained. In this dataset using the HarP protocol for segmentation, the mean number of hippocampal contours is ~37, meaning that eight or nine contours would reduce the outlining time to approximately one fourth of the full outlining time, comparable to dataset 1. From the Jaccard indices of Fig 7 it can be seen that the MCI group has slightly lower Jaccard indices than the CTRL group and the AD group has slightly lower indices than the MCI group. Overlap indices tend to be lower for smaller volumes. To determine to what extent the decrease in Jaccard indices in Fig 7 is a volume effect we plotted the volumes of manual segmentations against the observed Jaccard indices in Fig 8. In the same plot stacked histograms are shown to illustrate frequencies of volumes in specific groups. From the scatter plot it can be observed that Jaccard indices increase with hippocampal volume and that all three patient groups behave identically, i.e. that the volume difference drives the difference in Jaccard index.

### 3.3. Results for dataset 3

**Fig 9:**
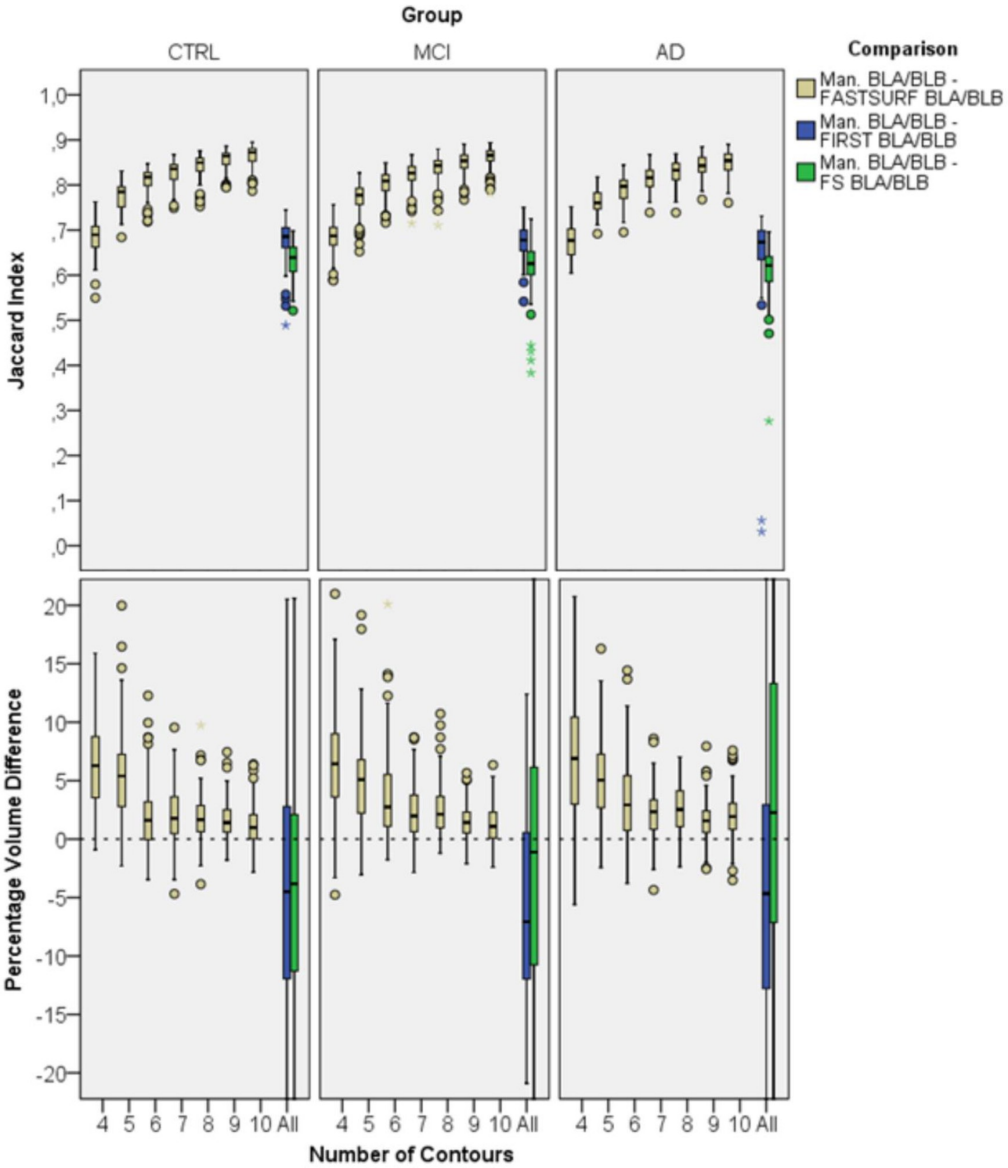
Agreement of FASTSURF and automatic segmentation methods with manual segmentations using BL scans. Left and right hippocampus segmentations were grouped together. Left boxplot shows Jaccard indices and the right boxplot PVDs. The small circle and the star sign are outliers defined by the SPSS software, with the star sign being a “far out” outlier.

For dataset 3 we obtained 280 hippocampus segmentations for 70 subjects with 4 MRIs at different time-points. Data of 10 MCI subjects were used for algorithm optimization and were therefore excluded from this analysis. We performed agreement (biased), robustness (unbiased) and atrophy (biased) analyses to assess FASTSURF’s performance. Fig 9 shows the biased Jaccard indices and PVDs comparing manual segmentations of the BL scans with corresponding FASTSURF segmentations for each diagnostic group. In both boxplots left and right hippocampus segmentations were grouped together. In the right part of each panel, the results for the automatic methods are shown. One can observe that FASTSURF segmentation with only five contours agree better with manual than fully automatic methods and with six contours PVDs are consistently close to the zero line.

**Fig 10:**
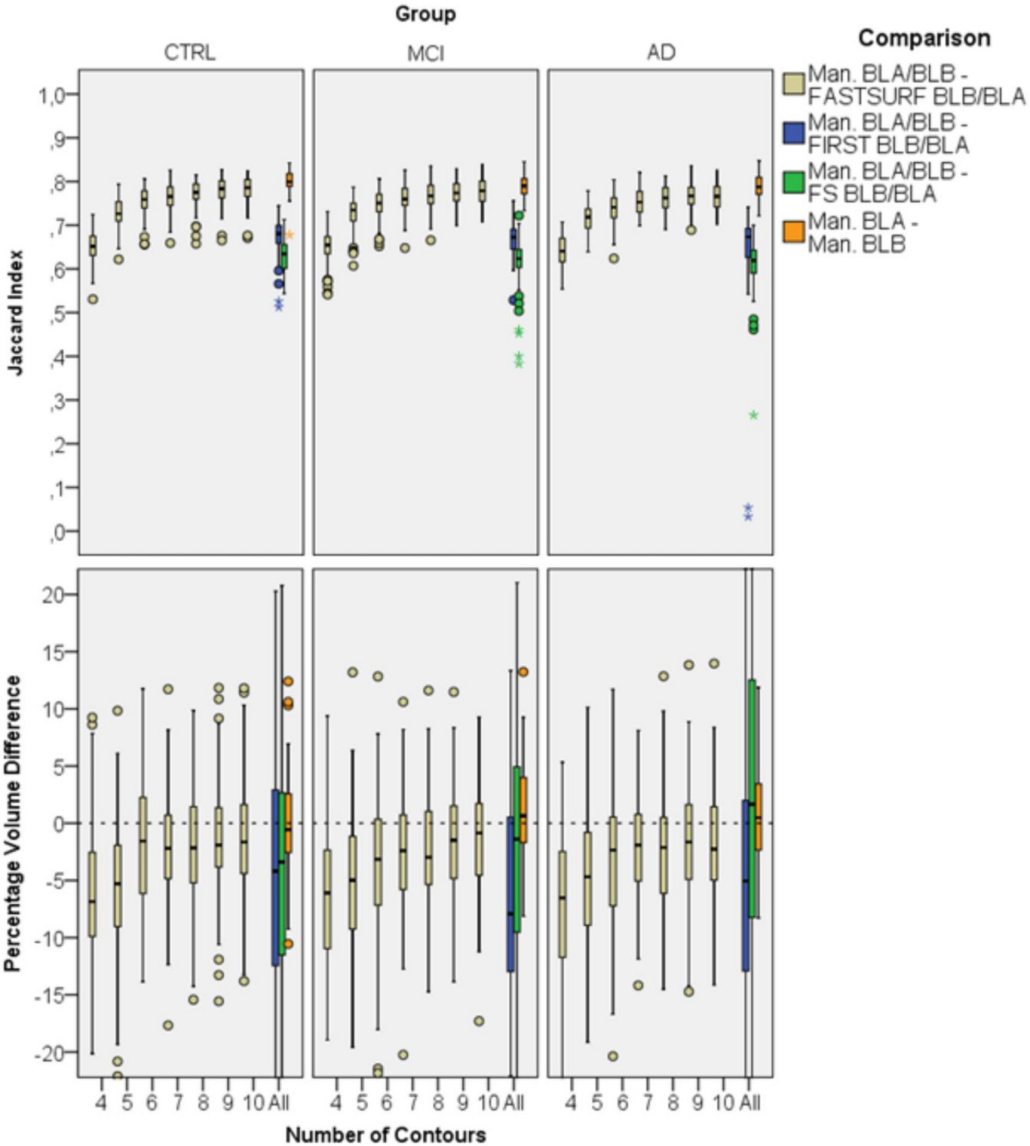
Robustness analysis of FASTSURF and automatic segmentation methods using BL scans. Left and right hippocampus segmentations were grouped together. Left boxplot shows Jaccard indices and the right boxplot PVDs. The orange boxes (left most boxes) illustrate the reproducibility of segmentation in BTB scans and gives a measure of the maximum possible level of accuracy. The small circle and the star sign are outliers defined by the SPSS software, with the star sign being a “far out” outlier.

Fig 10 presents the corresponding unbiased robustness analysis. Similar as for Fig 7 and Fig 9, it is visible in Fig 10 that results do not change much after a certain contour number threshold, i.e. for Fig 7 after eight contours and for Fig 9 and Fig 10 after six contours. The Jaccard indices of Fig 10 are slightly smaller than their biased variants and the PVD values are centred around zero for six contours and more. It is maintained that FASTSURF with only five contours performs better than the tested automatic methods. Also, Jaccard indices and PVDs for manual BTB hippocampus segmentations are presented, indicating the reproducibility of the manual observer. Manual hippocampus segmentation is often regarded as the “gold standard” [34,49], thus manual outline reproducibility represents a desirable level of accuracy to be reached. In study design, manual outline reproducibility is the maximum level of accuracy that can be reached with FASTSURF, because we extract contours from manual segmentations and FASTSURF segmentation follow the shape of these contours. Similar boxplots were obtained for M12 scans’ segmentations which can be viewed in the supplementary files (S1 and S2 Figs). S3 and S4 Tables display all corresponding mean and standard deviations.

The bias was quantified by subtracting unbiased results (Jacc_Unbiased_ and PVD_Unbiased_) shown in Fig 10 from the biased results (Jacc_Biased_ and PVD_Biased_) shown in Fig 9 as a function of input contours. As expected, for both BL and M12, the bias increases with increasing number of contours and ranges from 0.032(±0.0139) to 0.087(±0.0239) for the Jaccard indices and from -0.321(±2.5586)% to -2.477(±3.3234)% for PVDs.

With six or more contours, Jaccard indices and PVDs are relatively consistent–six contours would theoretically reduce segmentation time by approximately one third considering that the mean number of outlined contours for this dataset of ~20.

**Fig 11:**
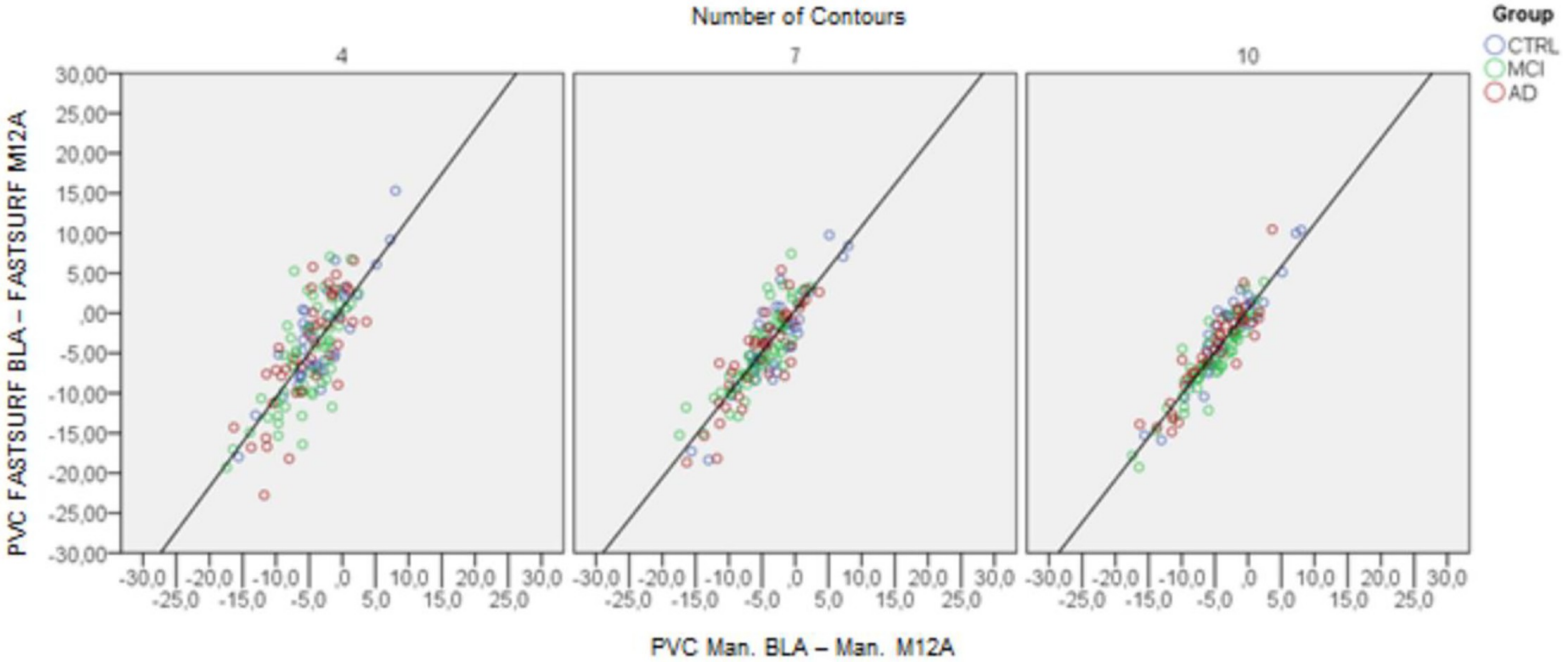
Scatter plots with atrophy rate measurements (PVC from baseline to follow-up) comparing FASTSURF and manual segmented hippocampi of the A scans from Dataset 3.

In Fig 11 three scatter plots show the correlation of hippocampal atrophy rates as determined by manual segmentations and FASTSURF using 4, 7 and 10 contours for the A scans’ hippocampi. Correlations (R^2^) for other numbers of input contours are given in Table 5. The last three lines in Table 5 present analogous correlations comparing atrophy measurements based on manual and FSL-FIRST, manual and FreeSurfer, and finally manually determined atrophy using A and the B scans.

**Table 5:** R^2^ values measured to compare atrophy rate measurements of A-scans’ manual hippocampus segmentations with automatic methods and FASTSURF.

Comparison	R^2^
Man. vs FASTSURF 4 Cont	0.594
Man. vs FASTSURF 5 Cont	0.541
Man. vs FASTSURF 6 Cont	0.599
Man. vs FASTSURF 7 Cont	0.752
Man. vs FASTSURF 8 Cont	0.733
Man. vs FASTSURF 9 Cont	0.807
Man. vs FASTSURF 10 Cont	0.845
Man. vs FIRST	0.024
Man. vs FS	0.015
Man. A vs Man. B	0.041

*Man*. Manual, *FS* FreeSurfer, *FASTSURF # Cont*. FASTSURF segmentations with # Contours

The correlation expectably increased with increasing number of contours. Atrophy rates derived from FASTSURF correlated consistently better with manually measured atrophy rates than atrophy rate measurements based on either automatic segmentation method. Even though this comparison is biased towards FASTSURF, the difference in R^2^ between automatic segmentation and FASTSURF is much larger than the estimated bias reported above. Similar results were obtained when using B-scans instead of A-scans.

## 4. Discussion

This study was performed to show the proof of concept of a novel semi-automatic hippocampus segmentation method (FASTSURF) which can substantially reduce segmentation time while maintaining high accuracy.

The novelty of FASTSURF is that it is entirely based on mesh processing procedures, i.e. image intensity, structural shape information or atlases are not needed. Therefore, we believe that FASTSURF is less prone to image noise or artefacts compared to intensity-based methods. Furthermore, the completion of a hippocampus given a sparse set of contours is computationally inexpensive and hippocampi are reconstructed within a second. The hippocampus is a thin seahorse-shaped structure which has geometrically more variation in shape than other subcortical brain structures or other soft tissue structures in the body. Since FASTSURF does not require specific anatomical a priori knowledge other than smoothness we expect that FASTSURF can also be used to outline different anatomical regions with similar or even better accuracy, depending on the shape of the structure.

Using simulated input extracted from different datasets we quantified the agreement to manual hippocampus segmentation by the Jaccard index and PVD measures. With FASTSURF we reached good accuracy with a Jaccard index of higher than 0.67 (equivalent to a Dice overlap of 0.8) by using only five contours for dataset 1 (μ = 0.75±0.035), seven contours for all groups in dataset 2 (μ_CTRL_ = 0.76±0.025, μ_MCI_ = 0.74±0.034, μ_AD_ = 0.72±0.043) and five contours for all groups in dataset 3 (Biased: μ_CTRL_ = 0.78±0.030, μ_MCI_ = 0.77±0.033, μ_AD_ = 0.76±0.026; Unbiased: μ_CTRL_ = 0.73±0.033, μ_MCI_ = 0.73±0.035, μ_AD_ = 0.72±0.031). Furthermore, as it can be seen from the Jaccard indices from dataset 3, the agreement to manual segmentation was considerably better than both tested automatic methods with only five contours for both biased and unbiased comparisons. Mean PVDs with five contours still seem to be quite high, ranging from 2.40(±3.67)-8.20(±3.71)% across data sets. PVDs improve considerably from seven contours onwards with mean PVDs ranging from 0.02(±2.40)–3.2(±3.40)% for the different data sets.

With dataset 3 we were also able to determine atrophy rates and compare atrophy rate measurements of FASTSURF, FreeSurfer and FSL-FIRST with manual segmentation. From Fig 11 and the R^2^ values of Table 5, it is evident that atrophy measurement using FASTSURF agrees more closely with atrophy derived from manual outlines than atrophy determined by either automatic segmentation methods. Visually inspecting Fig 11 and Table 5 suggests that using FASTSURF hippocampus segmentations with seven to ten input contours is sufficient with R^2^ values ranging from 0.75–0.85. Therefore, if this type of outlining protocol would be used, we recommend the use of seven contours as a practical compromise between accuracy and delineation time.

Most of our comparisons show very promising results in terms of accuracy of volume, Jaccard index and atrophy, but for part of the data sets they are biased. However, the unbiased robustness analysis performed with dataset 3 confirmed that FASTSURF segmentations agree better with manual segmentations than both automatic segmentation methods. Good and consistent overlap indices and PVDs were obtained by using six or more contours–our atrophy measurements suggest the need of seven or more contours. The robustness analysis indicates that slight variations of contour outlines does not affect the performance of the reconstruction method and that the bias is small. Therefore, our results suggest that these conclusions are equally valid for the data sets segmented with other protocols, but this needs to be confirmed in future studies.

The HarP protocol is the most modern and broadly accepted protocol in neuroscience, used to perform standardized and reproducible manual hippocampal segmentations [35]. In this study, HarP simulated contours were reconstructed with FASTSURF and compared to the manual counterpart segmentation. Results show high and consistent accuracy with eight or more contours–eight contours would reduce segmentation time by one fourth. This comparison is biased, but results of dataset 3 indicate that the bias is relatively small. We suggest that HarP can be combined with FASTSURF with minimum loss of accuracy, but this needs to be validated in future studies. Therefore, we conclude that FASTSURF would be very useful for efficient and reproducible hippocampus outlining. In radiotherapy, after delineating the hippocampus, a 5mm margin is placed around the hippocampus determining the region for dose sparing [10]. With FASTSURF we obtained high overlap results for hippocampi of dataset 1 with only five contours, indicating that this method can possibly be used for delineation in hippocampal sparing brain irradiation.

We emphasize that the completion of the hippocampus given a sparse delineation is computationally inexpensive and hippocampi are reconstructed within a second. Automatic segmentations, due to registration procedures of atlases, are usually computationally more expensive and it takes multiple minutes or hours to obtain a hippocampus segmentation. This leads to another advantage of FASTSURF because atlases, registration procedures, or parameter tweaking are not needed.

Compared to literature, we obtained similar overlap and PVD results for both automatic methods in comparison to manual segmentation [12,14,16–23,24–26]. Most of the literature mentions that automatic segmentation methods are comparable to manual hippocampus segmentation, i.e. show similar hippocampal volume trends for diagnostic groups, but they still need to improve to become as good as the gold standard. Recent papers even suggested that FreeSurfer might be used clinically for specific applications [23,24]. We showed that with FASTSURF, segmentations are consistently closer to manual hippocampus segmentations than FreeSurfer and FSL-FIRST without producing outliers. This suggests that FASTSURF is possibly closer to clinical implementation than automatic segmentations.

Comparison of FreeSurfer and FSL-FIRST with manual segmentations from dataset 3 might not be completely fair, because both automatic methods are trained with a different outlining protocol from the Center of Morphometric Analysis (CMA). The ANOVA volume analysis also indicates an overall outlining protocol difference with p-values lower than 0.005. However, with the post hoc ANOVA volume analysis we actually showed that BL left and right hippocampal volumes from FreeSurfer and manual segmentations were not significantly different (p = 0.341 and p = 0.070), but FSL-FIRST and FreeSurfer volumes were significantly different even though they were trained on the same outlining protocol (BL left: p = 0.070; BL right: p = 0.000009; M12 left: p = 0.030; M12 right: p = 0.000003). This indicates that at least on a volumetric level the outlining protocols are not very different. Extensive manual–automatic hippocampus segmentation analysis has been done previously, therefore we did not expand this outlining protocol investigation. Here, we merely demonstrate that FSL-FIRST and FreeSurfer hippocampus segmentations are less close to manual segmentations than FASTSURF segmentation, but for a completely unbiased comparison FreeSurfer and FSL-FIRST would have been trained with the same outlining protocol.

Furthermore, it would be interesting to compare FASTSURF to other automatic segmentation method such as multi-atlas/template-based segmentation methods [50,51], patch-based segmentation methods [52] or modern deep learning based methods as they emerge. In terms of segmentation results and segmentation speed the patch-based method seems very promising. In future studies, multi-atlas/template-based segmentation methods can be trained and tested with the manual segmentations from dataset 2 or 3 and finally, these methods can be compared to FASTSURF segmentations. Currently, the comparison to FSL-FIRST and FreeSurfer is the most important because these are the most used and tested publicly available segmentation methods.

Considering segmentation time reduction, we are not able to exactly predict how much time an observer would save for hippocampal segmentation, because this is a simulation study. As a rough estimate, one can take the number of contours taken for reconstruction, divide it by the mean number of total contours and multiply it by an estimated segmentation time for total hippocampus segmentation. As an example, if an expert rater takes ~2h to segment the left and right hippocampus outlining 36 slices, using our method the rater would only take ~30min if he/she outlines the hippocampus on 9 slices. Suggesting an optimal number of contours for accurate hippocampus reconstruction also depends on the desired level of accuracy. We think that with our method the number of contours can be at least reduced by half, if not by three quarters.

This study has two minor limitations. So far, only one contour on each slice is allowed to be outlined. This might not always be sufficient, because hippocampal atrophy can cause irregular hippocampal shapes leading to two or more contours per slice. Furthermore, if the hippocampus contains cavities that should be excluded from the hippocampal volume special precautions in the outlining software need to be implemented to account for such structures.

Another limitation of this study is that sparse segmentations were simulated from full manual segmentations. The present study was intended to demonstrate the proof of concept by providing initial validation. Future studies should produce true sparse delineations *de novo*, ideally including independent sparse delineations from multiple observers for a more complete validation. Furthermore, observers usually inspect neighbouring slices to outline the hippocampus. In theory, sparse segmentations could also be obtained by inspecting the neighbouring slices, which might slightly affect the delineation time.

FASTSURF is based on smooth interpolation and therefore it is, in its present form, not suited to delineate structures with irregular shapes such as tumours. However, for smooth structures such as the amygdala, thalamus, putamen or the caudate nucleus FASTSURF might work as well as for the hippocampus. Furthermore, manually selecting and including additional contours at inflection and high curvature points most probably improves FASTSURF’s accuracy for segmenting irregular shapes.

## 5. Conclusion

FASTSURF provides hippocampus outlines that are highly similar to completely manual segmentations and agree consistently better with manual segmentations than automatic segmentation methods (FSL-FIRST and FreeSurfer). Dependent on its implementation and the associated workflow, FASTSURF can reduce the time for expert observers to at least a half. Because in principle observers do not need to be retrained and because the method is computationally inexpensive, the proposed method is expected to be easily integrated into existing workflows. Future work needs to validate FASTSURF with partial segmentation performed by expert raters, which might lead to a possible usage of this method in the clinic.

## Supporting information

S1 Table The agreement analysis of FASTSURF with original hippocampus segmentations of dataset 1. (DOCX)

S2 Table Agreement analysis of FASTSURF with original hippocampus segmentations in groups for dataset 2. (DOCX)

S3 Table Agreement analysis of FASTSURF and automatic methods with manual hippocampus segmentations in groups for dataset 3. (DOCX)

S4 Table Robustness analysis of FASTSURF and automatic methods with manual hippocampus segmentations in groups for dataset 3. (DOCX)

S1 Fig Agreement analysis of FASTSURF and automatic methods with manual hippocampus segmentations using M12 scans. (TIF)

S2 Fig Robustness analysis of FASTSURF and automatic methods with manual hippocampus segmentations using M12 scans. (TIF)

S1 Supporting Information Dataset 1. (XLSX)

S2 Supporting Information Dataset 2. (XLSX)

S3 Supporting Information Dataset 3—Agreement analysis. (XLSX)

S4 Supporting Information Dataset 3—Robustness analysis. (XLSX)

S5 Supporting Information Dataset 3—Atrophy analysis. (XLSX)
