# Supplementary material for: FAst Segmentation Through SURface Fairing (FASTSURF): A novel semi-automatic hippocampus segmentation method
Source: PLoS One. 2019 Jan 18;14(1):e0210641. doi: 10.1371/journal.pone.0210641 (PMC6338359; doi:10.1371/journal.pone.0210641)
Supplement: S1 Table — (DOCX) [file pone.0210641.s001.docx]

| N. of Cont. |  | | Jaccard | | PVD | |
| --- | --- | --- | --- | --- | --- | --- |
|  | N | Mean | | STD | Mean | STD |
| 4 | 24 | .687 | | .0497 | -.098 | 5.2703 |
| 5 | 24 | .750 | | .0353 | 2.396 | 3.6663 |
| 6 | 24 | .783 | | .0324 | .746 | 1.7663 |
| 7 | 24 | .815 | | .0297 | .022 | 2.3980 |
| 8 | 24 | .840 | | .0253 | .285 | 1.7386 |
| 9 | 24 | .859 | | .0215 | .772 | 1.4246 |
| 10 | 24 | .870 | | .0262 | -.372 | 1.1288 |

*N. of Cont.* Number of Contours, *PVD* Percentage Volume Difference, *STD* Standard Deviation
