# Supplementary material for: FAst Segmentation Through SURface Fairing (FASTSURF): A novel semi-automatic hippocampus segmentation method
Source: PLoS One. 2019 Jan 18;14(1):e0210641. doi: 10.1371/journal.pone.0210641 (PMC6338359; doi:10.1371/journal.pone.0210641)
Supplement: S2 Table — (DOCX) [file pone.0210641.s002.docx]

| N. of Cont. | Group |  | Jaccard | | PVD | |
| --- | --- | --- | --- | --- | --- | --- |
|  |  | N | Mean | STD | Mean | STD |
| 4 | AD | 90 | .526 | .0577 | 16.322 | 6.9249 |
|  | CTRL | 88 | .566 | .0393 | 14.521 | 5.0772 |
|  | MCI | 92 | .533 | .0540 | 16.703 | 5.4952 |
| 5 | AD | 90 | .650 | .0374 | 7.704 | 3.3577 |
|  | CTRL | 88 | .692 | .0308 | 7.651 | 3.3528 |
|  | MCI | 92 | .662 | .0371 | 8.201 | 3.7148 |
| 6 | AD | 90 | .687 | .0381 | 6.163 | 4.1511 |
|  | CTRL | 88 | .732 | .0250 | 4.661 | 3.6872 |
|  | MCI | 92 | .708 | .0389 | 5.942 | 3.8069 |
| 7 | AD | 90 | .718 | .0427 | 3.249 | 3.3976 |
|  | CTRL | 88 | .766 | .0250 | 1.627 | 3.0434 |
|  | MCI | 92 | .742 | .0335 | 2.833 | 2.9815 |
| 8 | AD | 90 | .746 | .0356 | 1.240 | 3.1072 |
|  | CTRL | 88 | .790 | .0254 | .750 | 2.5596 |
|  | MCI | 92 | .766 | .0301 | 1.731 | 2.4134 |
| 9 | AD | 90 | .765 | .0381 | 1.268 | 2.3423 |
|  | CTRL | 88 | .809 | .0241 | .294 | 1.8273 |
|  | MCI | 92 | .782 | .0306 | .965 | 2.2414 |
| 10 | AD | 90 | .781 | .0360 | 1.115 | 2.3853 |
|  | CTRL | 88 | .824 | .0216 | .610 | 1.3903 |
|  | MCI | 92 | .801 | .0292 | .570 | 1.7294 |
| 11 | AD | 90 | .792 | .0354 | 1.074 | 2.0583 |
|  | CTRL | 88 | .833 | .0208 | .399 | 1.4250 |
|  | MCI | 92 | .814 | .0274 | .731 | 1.7009 |
| 12 | AD | 90 | .804 | .0328 | .951 | 1.9566 |
|  | CTRL | 88 | .842 | .0197 | .380 | 1.1379 |
|  | MCI | 92 | .823 | .0278 | .667 | 1.5282 |
| 13 | AD | 90 | .813 | .0313 | .870 | 1.5382 |
|  | CTRL | 88 | .849 | .0186 | .473 | 1.1178 |
|  | MCI | 92 | .833 | .0262 | .533 | 1.1759 |
| 14 | AD | 90 | .822 | .0316 | .602 | 1.5641 |
|  | CTRL | 88 | .855 | .0182 | .449 | 1.2040 |
|  | MCI | 92 | .840 | .0250 | .306 | 1.2182 |
| 15 | AD | 90 | .829 | .0280 | .420 | 1.0822 |
|  | CTRL | 88 | .862 | .0167 | .441 | .9092 |
|  | MCI | 92 | .845 | .0266 | .466 | 1.1060 |
| 16 | AD | 90 | .835 | .0263 | .811 | 1.2448 |
|  | CTRL | 88 | .868 | .0175 | .585 | .8968 |
|  | MCI | 92 | .851 | .0239 | .478 | 1.1247 |
| 17 | AD | 90 | .839 | .0291 | .681 | 1.1923 |
|  | CTRL | 88 | .872 | .0172 | .249 | .8842 |
|  | MCI | 92 | .856 | .0229 | .224 | .9689 |
| 18 | AD | 90 | .844 | .0293 | .832 | 1.1895 |
|  | CTRL | 88 | .877 | .0160 | .173 | .7776 |
|  | MCI | 92 | .861 | .0231 | .397 | 1.0099 |

*N. of Cont.* Number of Contours, *PVD* Percentage Volume Difference, *STD* Standard Deviation, *CTRL* Controls, *MCI* Mild Cognitive Impairment, *AD* Alzheimer’s Disease
