# Supplementary material for: FAst Segmentation Through SURface Fairing (FASTSURF): A novel semi-automatic hippocampus segmentation method
Source: PLoS One. 2019 Jan 18;14(1):e0210641. doi: 10.1371/journal.pone.0210641 (PMC6338359; doi:10.1371/journal.pone.0210641)
Supplement: S3 Table — (DOCX) [file pone.0210641.s003.docx]

| N. of Cont. | Comparison | Group |  | | Jaccard | | PVD | |
| --- | --- | --- | --- | --- | --- | --- | --- | --- |
|  |  |  | N | Mean | | STD | Mean | STD |
| 4 | Man. BLA/BLB - FASTSURF BLA/BLB | AD | 80 | .675 | | ,0345 | 7,217 | 5,6270 |
|  |  | CTRL | 80 | .687 | | ,0383 | 6,248 | 3,7123 |
|  |  | MCI | 120 | .687 | | ,0358 | 6,688 | 4,7401 |
|  | Man. M12A/M12B - FASTSURF M12A/M12B | AD | 80 | .661 | | ,0424 | 6,927 | 5,5458 |
|  |  | CTRL | 80 | .691 | | ,0380 | 5,913 | 4,2492 |
|  |  | MCI | 120 | .681 | | ,0373 | 7,144 | 4,5478 |
| 5 | Man. BLA/BLB - FASTSURF BLA/BLB | AD | 80 | .761 | | ,0264 | 5,113 | 3,6093 |
|  |  | CTRL | 80 | .776 | | ,0305 | 5,536 | 3,9984 |
|  |  | MCI | 120 | .770 | | ,0331 | 4,920 | 3,9377 |
|  | Man. M12A/M12B - FASTSURF M12A/M12B | AD | 80 | .751 | | ,0364 | 4,949 | 3,7927 |
|  |  | CTRL | 80 | .779 | | ,0260 | 5,454 | 3,3935 |
|  |  | MCI | 120 | .767 | | ,0327 | 4,939 | 3,7318 |
| 6 | Man. BLA/BLB - FASTSURF BLA/BLB | AD | 80 | .791 | | ,0292 | 3,141 | 3,7728 |
|  |  | CTRL | 80 | .809 | | ,0300 | 1,967 | 3,0244 |
|  |  | MCI | 120 | .802 | | ,0303 | 3,665 | 3,8245 |
|  | Man. M12A/M12B - FASTSURF M12A/M12B | AD | 80 | .781 | | ,0342 | 3,632 | 4,2064 |
|  |  | CTRL | 80 | .807 | | ,0259 | 2,507 | 2,9493 |
|  |  | MCI | 120 | .798 | | ,0335 | 3,579 | 3,8733 |
| 7 | Man. BLA/BLB - FASTSURF BLA/BLB | AD | 80 | .815 | | ,0243 | 2,122 | 2,2001 |
|  |  | CTRL | 80 | .827 | | ,0262 | 2,012 | 2,4649 |
|  |  | MCI | 120 | .822 | | ,0279 | 2,363 | 2,4479 |
|  | Man. M12A/M12B - FASTSURF M12A/M12B | AD | 80 | .804 | | ,0357 | 2,454 | 2,5763 |
|  |  | CTRL | 80 | .827 | | ,0236 | 2,191 | 2,3789 |
|  |  | MCI | 120 | .821 | | ,0272 | 2,079 | 2,4889 |
| 8 | Man. BLA/BLB - FASTSURF BLA/BLB | AD | 80 | .828 | | ,0268 | 2,562 | 2,1011 |
|  |  | CTRL | 80 | .841 | | ,0282 | 1,901 | 2,1132 |
|  |  | MCI | 120 | .836 | | ,0303 | 2,503 | 2,2523 |
|  | Man. M12A/M12B - FASTSURF M12A/M12B | AD | 80 | .819 | | ,0293 | 2,872 | 2,4295 |
|  |  | CTRL | 80 | .842 | | ,0226 | 2,388 | 1,9259 |
|  |  | MCI | 120 | .833 | | ,0329 | 2,762 | 2,2274 |
| 9 | Man. BLA/BLB - FASTSURF BLA/BLB | AD | 80 | .842 | | ,0240 | 1,576 | 1,8838 |
|  |  | CTRL | 80 | .856 | | ,0225 | 1,631 | 1,8225 |
|  |  | MCI | 120 | .849 | | ,0242 | 1,471 | 1,5862 |
|  | Man. M12A/M12B - FASTSURF M12A/M12B | AD | 80 | .833 | | ,0349 | 1,599 | 1,9441 |
|  |  | CTRL | 80 | .856 | | ,0199 | 1,427 | 1,8519 |
|  |  | MCI | 120 | .849 | | ,0236 | 1,446 | 1,5056 |
| 10 | Man. BLA/BLB - FASTSURF BLA/BLB | AD | 80 | .848 | | ,0277 | 2,126 | 2,0728 |
|  |  | CTRL | 80 | .863 | | ,0247 | 1,251 | 1,9124 |
|  |  | MCI | 120 | .860 | | ,0250 | 1,301 | 1,7111 |
|  | Man. M12A/M12B - FASTSURF M12A/M12B | AD | 80 | .846 | | ,0297 | 1,748 | 1,5655 |
|  |  | CTRL | 80 | .864 | | ,0219 | 1,240 | 1,7480 |
|  |  | MCI | 120 | .858 | | ,0268 | 1,619 | 1,7297 |
| All | Man. BLA/BLB - FIRST BLA/BLB | AD | 80 | .650 | | ,1072 | -4,710 | 13,7202 |
|  |  | CTRL | 80 | .677 | | ,0469 | -4,317 | 10,8961 |
|  |  | MCI | 120 | .675 | | ,0348 | -5,724 | 8,4339 |
|  | Man. BLA/BLB –  FS BLA/BLB | AD | 80 | .609 | | ,0586 | 2,175 | 13,8728 |
|  |  | CTRL | 80 | .633 | | ,0403 | -4,569 | 10,7623 |
|  |  | MCI | 120 | .619 | | ,0521 | -1,574 | 12,3219 |
|  | Man. M12A/M12B - FIRST M12A/M12B | AD | 80 | .633 | | ,1378 | -5,608 | 14,5948 |
|  |  | CTRL | 80 | .679 | | ,0440 | -6,225 | 9,9698 |
|  |  | MCI | 120 | .670 | | ,0383 | -7,389 | 9,0893 |
|  | Man. M12A/M12B - FS M12A/M12B | AD | 80 | .592 | | ,0873 | 3,825 | 14,0565 |
|  |  | CTRL | 80 | .635 | | ,0355 | -6,759 | 11,0298 |
|  |  | MCI | 120 | .618 | | ,0686 | -2,550 | 13,1361 |

*N. of Cont.* Number of Contours, *PVD* Percentage Volume Difference, *STD* Standard Deviation, *Man.* Manual, *FS* FreeSurfer, *BLA/B* Baseline A/B, *M12A/B* Month-12 A/B, *CTRL* Controls, *MCI* Mild Cognitive Impairment, *AD* Alzheimer’s Disease
