# Supplementary material for: FAst Segmentation Through SURface Fairing (FASTSURF): A novel semi-automatic hippocampus segmentation method
Source: PLoS One. 2019 Jan 18;14(1):e0210641. doi: 10.1371/journal.pone.0210641 (PMC6338359; doi:10.1371/journal.pone.0210641)
Supplement: S4 Table — (DOCX) [file pone.0210641.s004.docx]

| N. of Cont. | Comparison | Group |  | | Jaccard | | PVD | |
| --- | --- | --- | --- | --- | --- | --- | --- | --- |
|  |  |  | N | Mean | | STD | Mean | STD |
| 4 | Man. BLA/BLB - FASTSURF BLB/BLA | AD | 80 | .642 | | ,0355 | -7,213 | 6,9611 |
|  |  | CTRL | 80 | .651 | | ,0351 | -6,245 | 5,9628 |
|  |  | MCI | 120 | .654 | | ,0373 | -6,685 | 6,3025 |
|  | Man. M12A/M12B - FASTSURF M12B/M12A | AD | 80 | .625 | | ,0416 | -6,924 | 7,3501 |
|  |  | CTRL | 80 | .659 | | ,0401 | -5,912 | 5,1468 |
|  |  | MCI | 120 | .647 | | ,0395 | -7,142 | 5,7788 |
| 5 | Man. BLA/BLB - FASTSURF BLB/BLA | AD | 80 | .717 | | ,0308 | -5,109 | 6,1961 |
|  |  | CTRL | 80 | .728 | | ,0332 | -5,533 | 6,1184 |
|  |  | MCI | 120 | .727 | | ,0353 | -4,918 | 5,7499 |
|  | Man. M12A/M12B - FASTSURF M12B/M12A | AD | 80 | .703 | | ,0396 | -4,946 | 5,7197 |
|  |  | CTRL | 80 | .736 | | ,0288 | -5,453 | 4,6697 |
|  |  | MCI | 120 | .722 | | ,0348 | -4,937 | 5,1601 |
| 6 | Man. BLA/BLB - FASTSURF BLB/BLA | AD | 80 | .737 | | ,0354 | -3,139 | 6,3610 |
|  |  | CTRL | 80 | .754 | | ,0344 | -1,966 | 5,6482 |
|  |  | MCI | 120 | .748 | | ,0319 | -3,662 | 6,3410 |
|  | Man. M12A/M12B - FASTSURF M12B/M12A | AD | 80 | .723 | | ,0396 | -3,631 | 6,5261 |
|  |  | CTRL | 80 | .757 | | ,0296 | -2,506 | 4,2959 |
|  |  | MCI | 120 | .744 | | ,0390 | -3,578 | 5,3942 |
| 7 | Man. BLA/BLB - FASTSURF BLB/BLA | AD | 80 | .755 | | ,0296 | -2,121 | 4,9684 |
|  |  | CTRL | 80 | .765 | | ,0320 | -2,011 | 5,1435 |
|  |  | MCI | 120 | .760 | | ,0312 | -2,362 | 5,1122 |
|  | Man. M12A/M12B - FASTSURF M12B/M12A | AD | 80 | .739 | | ,0406 | -2,453 | 5,0496 |
|  |  | CTRL | 80 | .770 | | ,0284 | -2,191 | 3,7142 |
|  |  | MCI | 120 | .758 | | ,0339 | -2,078 | 4,4780 |
| 8 | Man. BLA/BLB - FASTSURF BLB/BLA | AD | 80 | .758 | | ,0322 | -2,561 | 5,1076 |
|  |  | CTRL | 80 | .772 | | ,0311 | -1,900 | 5,1537 |
|  |  | MCI | 120 | .767 | | ,0331 | -2,502 | 5,0352 |
|  | Man. M12A/M12B - FASTSURF M12B/M12A | AD | 80 | .747 | | ,0380 | -2,870 | 4,8118 |
|  |  | CTRL | 80 | .779 | | ,0255 | -2,387 | 3,4613 |
|  |  | MCI | 120 | .763 | | ,0377 | -2,761 | 3,8486 |
| 9 | Man. BLA/BLB - FASTSURF BLB/BLA | AD | 80 | .766 | | ,0325 | -1,575 | 5,0296 |
|  |  | CTRL | 80 | .779 | | ,0307 | -1,630 | 5,1041 |
|  |  | MCI | 120 | .773 | | ,0300 | -1,471 | 4,3936 |
|  | Man. M12A/M12B - FASTSURF M12B/M12A | AD | 80 | .754 | | ,0422 | -1,598 | 4,6366 |
|  |  | CTRL | 80 | .787 | | ,0259 | -1,426 | 3,6302 |
|  |  | MCI | 120 | .772 | | ,0327 | -1,446 | 3,7972 |
| 10 | Man. BLA/BLB - FASTSURF BLB/BLA | AD | 80 | .766 | | ,0312 | -2,125 | 5,0632 |
|  |  | CTRL | 80 | .782 | | ,0301 | -1,250 | 5,1092 |
|  |  | MCI | 120 | .778 | | ,0304 | -1,300 | 4,6359 |
|  | Man. M12A/M12B - FASTSURF M12B/M12A | AD | 80 | .759 | | ,0379 | -1,747 | 4,6049 |
|  |  | CTRL | 80 | .788 | | ,0269 | -1,240 | 3,2520 |
|  |  | MCI | 120 | .776 | | ,0347 | -1,619 | 3,6578 |
| All | Man. BLA –  Man. BLB | AD | 40 | .788 | | ,0300 | 1,020 | 4,3994 |
|  |  | CTRL | 40 | .797 | | ,0299 | ,171 | 4,8439 |
|  |  | MCI | 60 | .790 | | ,0279 | 1,136 | 4,2214 |
|  | Man. BLA/BLB - FIRST BLB/BLA | AD | 80 | .648 | | ,1070 | -5,290 | 13,9232 |
|  |  | CTRL | 80 | .673 | | ,0419 | -4,548 | 10,8048 |
|  |  | MCI | 120 | .670 | | ,0356 | -6,000 | 8,5629 |
|  | Man. BLA/BLB –  FS BLB/BLA | AD | 80 | .607 | | ,0618 | 2,175 | 13,9947 |
|  |  | CTRL | 80 | .631 | | ,0367 | -4,567 | 10,6207 |
|  |  | MCI | 120 | .617 | | ,0509 | -1,574 | 12,2955 |
|  | Man. M12A –  Man. M12B | AD | 40 | .776 | | ,0377 | ,490 | 4,3248 |
|  |  | CTRL | 40 | .802 | | ,0254 | -,070 | 2,8991 |
|  |  | MCI | 60 | .787 | | ,0334 | -1,298 | 3,2701 |
|  | Man. M12A/M12B - FIRST M12B/M12A | AD | 80 | .627 | | ,1350 | -6,617 | 15,8796 |
|  |  | CTRL | 80 | .676 | | ,0450 | -6,366 | 9,9425 |
|  |  | MCI | 120 | .667 | | ,0382 | -7,826 | 9,4764 |
|  | Man. M12A/M12B - FS M12B/M12A | AD | 80 | .588 | | ,0834 | 3,821 | 14,1814 |
|  |  | CTRL | 80 | .630 | | ,0351 | -6,759 | 11,0290 |
|  |  | MCI | 120 | .615 | | ,0695 | -2,554 | 13,1071 |

*N. of Cont.* Number of Contours, *PVD* Percentage Volume Difference, *STD* Standard Deviation, *Man.* Manual, *FS* FreeSurfer, *BLA/B* Baseline A/B, *M12A/B* Month-12 A/B, *CTRL* Controls, *MCI* Mild Cognitive Impairment, *AD* Alzheimer’s Disease
