# Supplementary figures and images for: FAst Segmentation Through SURface Fairing (FASTSURF): A novel semi-automatic hippocampus segmentation method
Source: PLoS One. 2019 Jan 18;14(1):e0210641. doi: 10.1371/journal.pone.0210641 (PMC6338359; doi:10.1371/journal.pone.0210641)

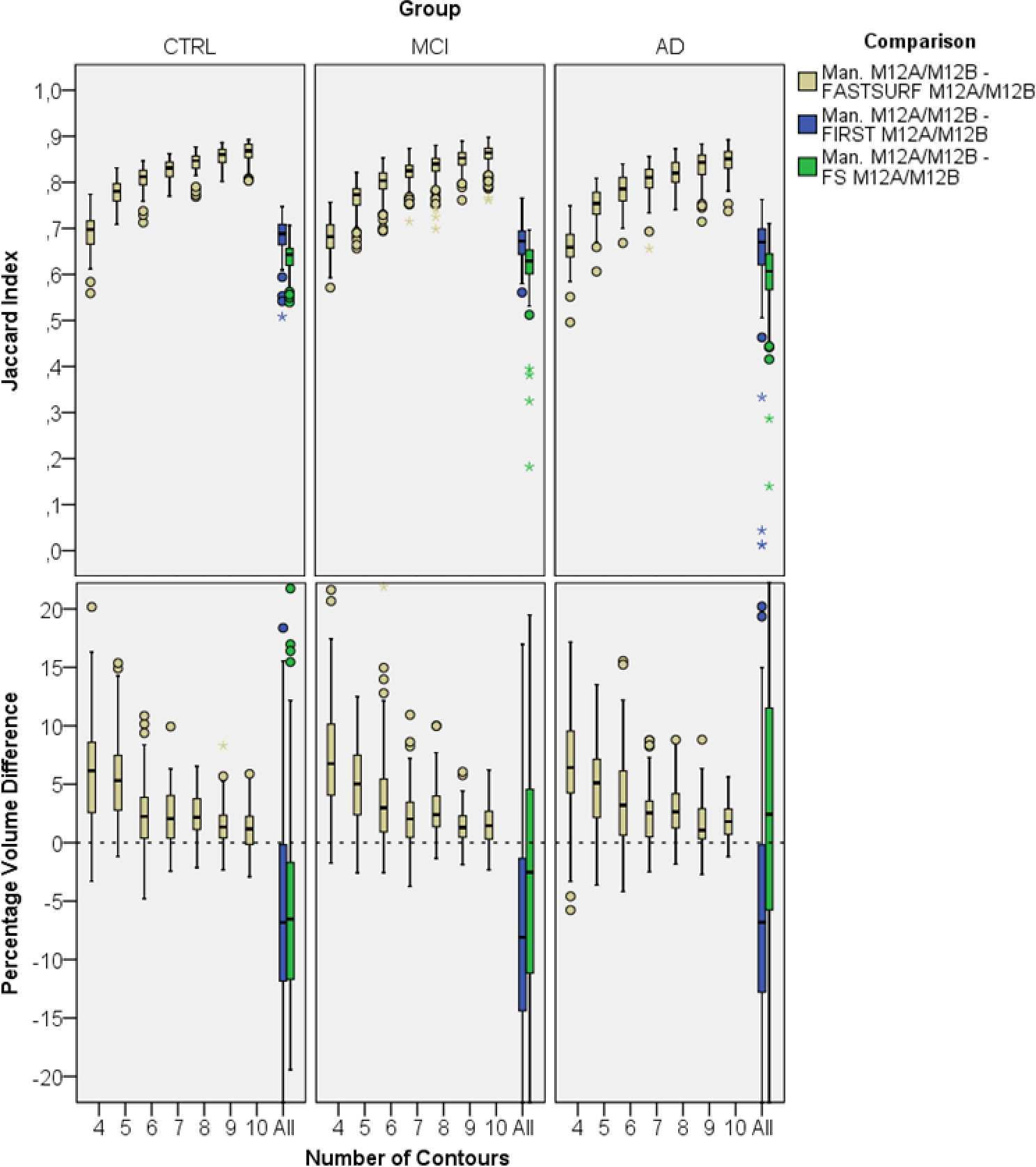

Supplement: S1 Fig — (TIF) [file pone.0210641.s005.tif]

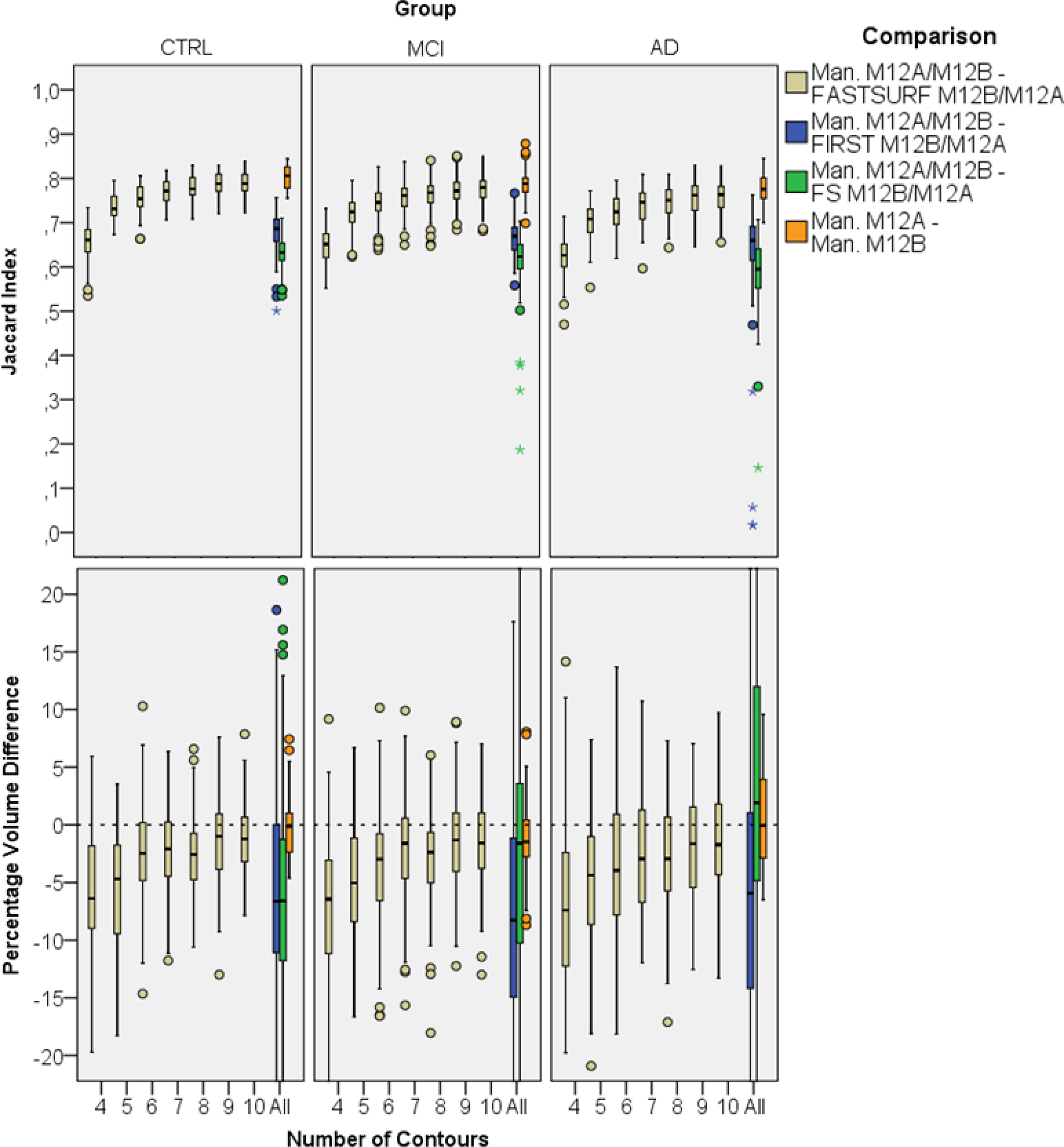

Supplement: S2 Fig — (TIF) [file pone.0210641.s006.tif]
